# Role of Plasmonic Antenna in Hot Carrier-Driven Reactions on Bimetallic Nanostructures

**DOI:** 10.1021/acs.jpcc.3c06520

**Published:** 2023-11-09

**Authors:** Zhandong Li, Joel Rigor, Sadaf Ehtesabi, Siddhi Gojare, Stephan Kupfer, Stefanie Gräfe, Nicolas Large, Dmitry Kurouski

**Affiliations:** † Department of Biochemistry and Biophysics, Texas A&M University, College Station, Texas 77843, United States; ‡ Department of Physics and Astronomy, The University of Texas at San Antonio, San Antonio, Texas 78249, United States; § Institute of Physical Chemistry and Abbe Center of Photonics, Friedrich Schiller University Jena, Helmholtzweg 4, 07743 Jena, Germany; ∥ The Institute for Quantum Science and Engineering, Texas A&M University, College Station, Texas 77843, United States

## Abstract

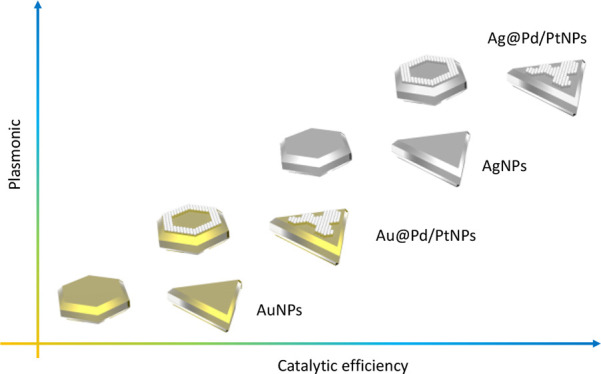

Noble metal nanostructures can efficiently harvest electromagnetic radiation, which, in turn, is used to generate localized surface plasmon resonances. Surface plasmons decay, producing hot carriers, that is, short-lived species that can trigger chemical reactions on metallic surfaces. However, noble metal nanostructures catalyze only a very small number of chemical reactions. This limitation can be overcome by coupling such nanostructures with catalytic-active metals. Although the role of such catalytically active metals in plasmon-driven catalysis is well-understood, the mechanistics of a noble metal antenna in such chemistry remains unclear. In this study, we utilize tip-enhanced Raman spectroscopy, an innovative nanoscale imaging technique, to investigate the rates and yields of plasmon-driven reactions on mono- and bimetallic gold- and silver-based nanostructures. We found that silver nanoplates (AgNPs) demonstrate a significantly higher yield of 4-nitrobenzenehtiol to *p*,*p*′-dimercaptoazobisbenzene (DMAB) reduction than gold nanoplates (AuNPs). We also observed substantially greater yields of DMAB on silver–platinum and silver–palladium nanoplates (Ag@PtNPs and Ag@PdNPs) compared to their gold analogues, Au@PtNPs and Au@PdNPs. Furthermore, Ag@PtNPs exhibited enhanced reactivity in 4-mercatophenylmethanol to 4-mercaptobenzoic acid oxidation compared to Au@PtNPs. These results showed that silver-based bimetallic nanostructures feature much greater reactivity compared to their gold-based analogues.

## Introduction

When noble metal nanostructures are illuminated by light, coherent oscillations of the conduction electrons, which are also known as localized surface plasmon resonances (LSPRs), can be observed.^1−6^ LSPRs can dissipate producing heat or decay into hot carriers, short-living and highly energetic species.^7,8^ Hot carriers can be injected through direct or indirect charge transfer into orbitals of molecules present in the close proximity to the metallic surfaces.^9−12^ This triggers chemical transformation in such molecular species.^7,13−15^ A growing body of evidence suggests that hot carriers have unequal rates of dissipation.^16,17^ As a result, species with “slower” transfer rates between a nanostructure and the surrounding medium accumulate on the nanostructures generating an electrostatic potential which can be used to modify rates of chemical reactions.^18,19^ The electrostatic potential can be altered by light intensity allowing for the direct and precise modulation of the chemical reactivity on noble metal nanosturctures.^20−25^

Coupling of plasmonic and catalytic-active metals, such as platinum (Pt) and palladium (Pd), in one nanostructure or a reactor-antenna system allows for a substantial expansion in the field of plasmon-driven chemistry.^26^ In this case, noble metal nanostructures generate LSPRs that are passed onto catalytic metals, where plasmon-driven chemistry takes place.^10,27^ Experimental results reported by our and other research groups showed that catalytic metals determine the selectivity of chemical transformations in such bimetallic systems.^18,28−36^ For instance, we found that 4-mercatophenylmethanol (4-MPM) can be oxidized into 4-mercaptobenzoic acid (4-MBA) only if Pt was present on the surface of gold nanoplates (AuNPs).^33^ In the absence of Pt, AuNPs were able to convert 4-MPM only into thiophenol (TP).^33,37^ We also found that gold–palladium nanoplates (Au@PdNPs) could be used to oxidize 3- and 4-MPM into 3- and 4-MBA, respectively, whereas their monometallic analogues (AuNPs) were capable of only decarboxylating 4-MBA into TP.^33,38^ Wang and co-workers discovered that gold–palladium Au@Pd nanoparticles could perform a plasmon-enhanced Suzuki coupling reaction demonstrating a 2-fold increase in the reaction rate and yield compared to the corresponding monometallic nanostructures.^15^ The researchers reported provided these Au@Pd nanoparticles compared to the rate and yield of corresponding monometallic nanostructures. Furthermore, the Halas group demonstrated that copper−ruthenium (Cu@Ru) nanostructures could be used for light-driven dry reforming of methane with carbon dioxide, a reaction that yields syngas.^14^ Lou et al. observed that Au@Pt nanoprisms could be utilized to generate molecular hydrogen, while various classes of platinum–gold nanoprisms, including Pt-covered, Pt-edged, and Pt-tipped Au nanoprisms, were investigated.^16^ It was found that Pt-edged bimetallic nanostructures led to nearly 5 times more efficient hydrogen generation compared to Pt-covered and Pt-tipped platinum–gold nanoprisms. These findings suggest that the catalytic efficiency of bimetallic nanostructures also depends on their nanoscale structural organization, which remains poorly understood.

A growing body of evidence shows that tip-enhanced Raman spectroscopy (TERS), a modern analytical technique that demonstrates Ångström spatial resolution,^39−41^ can be used to investigate a plasmon-driven process at the nanoscale. In TERS, the metal or metalized scanning probe is positioned within a few Ångströms from the sample surface.^39−41^ If such a probe is illuminated by light, LSPRs generated at the tip apex produce an electric field that is compressed to a pico-cavity.^42^ This local electric field enhances the Raman scattering from molecules present on the surface by a factor of 10^4^–10^6^, allowing for single molecule detection.^43^ Furthermore, the same electric field can also be used to trigger chemical transformations in the molecular analytes present on the surface. Thus, TERS can be used to (i) trigger and (ii) visualize plasmon-driven chemistry at the nanoscale.^44,45^

Using TERS, Li and co-workers found that the edges and corners of AuNPs exhibited significantly greater reactivity in plasmon-driven 4-nitrobenzenehtiol (4-NBT) to *p,p*′-dimercaptoazobisbenzene (DMAB) reduction compared to surfaces of these nanostructures.^18,25,46^ It was also shown that plasmon-driven reduction of 4-NBT on AuNPs yields only DMAB, whereas the same reduction reaction on Au@PdNPs results in the formation of both DMAB and 4-aminothiophenol (4-ATP).^47^ Datta’s group recently used TERS to capture the buckling distortions in silicene, whereas El-Khoury’s group investigated spatial variations in optical fields on the surface of Ag nanoparticles using this highly sensitive analytical approach.^31^ It was determined that AgNP edges exhibit higher signals compared to their center. A similar plasmonic effect has also been observed on the edges of silver nanowires. Using ultrahigh vacuum TERS, Ren’s group investigated the edge effects on the submonolayer of Pd on the Au surface.^32^ Using phenyl isocyanide (PIC) as a molecular reporter, the researchers showed that in TERS spectra collected at Pd edges, the C≡N vibrational modes of PIC were red-shifted by 60 cm^–1^ relative to molecules located on the Pd terrace.^30^ These results indicated that molecules located at Pd edges display a higher reactivity relative to that of Pd atoms in terraces.

Although the role of catalytic metals in plasmon-driven chemistry on bimetallic nanostructures is well-understood, there is very little, if anything, known about the role of the catalytic “antenna” in such systems. To end this, we measured the rates and yield of plasmon-driven reactions on Au- and Ag-based monometallic and bimetallic nanoplates. Furthermore, electrodynamic as well as quantum chemical simulations were performed in order to interpret the experimental observations.

## Methods

### Chemicals

Gold(III) chloride trihydrate (HAuCl_4_·3H_2_O, 99.9%), palladium(II) chloride solution (H_2_PdCl_4_), chloroplatinic acid solution (H_2_PtCl_6_), hexadecyltrimethylammonium bromide (CTAB, 99%), 4-nitrothiolphenol (4-NTP), 4-MBA, 2-nitro-5-sulfanylbenzoic acid, sodium hydroxide (NaOH, 98%), potassium iodide (KI, 99%), l-ascorbic acid (AA, 99%), and sodium borohydride (NaBH_4_, 99%) were purchased from Sigma-Aldrich (St. Louis, MO). Sodium citrate dihydrate (Na-Cit, 99%) was purchased from Fisher scientific (Waltham, MA). Ethanol was purchased from Decon Laboratories (King of Prussia, PA). Silver nitrate (AgNO_3_) and poly(vinylpyrrolidone) (PVP, *M*_W_ ≈ 29,000, 0.7 mM in terms of the repeating unit) were purchased from Aldrich. All chemicals were used as received without purification.

### Preparation of AuNPs, Au@PdNPs, and Au@PtNPs

The AuNPs were prepared first by seed-mediated growth method followed by isotropical growth. A gold seed solution was first prepared by adding 1 mL of 0.01 M HAuCl_4_ and 1 mL of 0.01 M Na-Cit solutions into 36 mL of water. Then, under vigorous stirring, 1 mL of 0.1 M ice-cold NaBH_4_ solution was introduced and stirred for 2 min. Next, the mixture solution was kept at room temperature without disturbing and aged for 2–6 h. AuNP seed solution with ∼15 nm thickness with a triangle or hexagonal shape was then prepared by three-step seed-mediated growth of the Au seeds after aging. Briefly, growing solutions 1, 2, and 3 were prepared using the following methods. First, growing solutions 1 and 2 were prepared by the same method where 0.25 mL of 0.01 M HAuCl_4_, 0.05 mL of 0.1 M NaOH, 0.05 mL of 0.01 M KI, and 0.05 mL of 0.1 M AA were added into 9 mL of 0.05 M CTAB solution by order. Then, the third growing solution was prepared similarly by introducing 2.5 mL of 0.01 M AuCl_4_, 0.5 mL of 0.1 M NaOH, 0.5 mL of 0.01 M KI, and 0.5 mL of 0.1 M AA into 90 mL of 0.05 M CTAB solution. Subsequently, 1 mL of the Au seed stocking solution was added into growing solution 1, followed by gentle shaking for 5 s. Then, 1 mL of the Au seeds and growing solution 1 mixture were added to growth solution 2, followed by gently shaking for another 5 s. Finally, all of the Au seeds and growth solution 1 and 2 mixtures were added to the growing solution 3 followed by gently shaking for 5 s. The final solution was then kept at room temperature overnight without any disturbance. Then, the ∼15 nm thick AuNP seeds were collected by precipitating at 5000 rpm for 2 min and dissolved in 5 mL of CTAB solution for the next step of growth. The thicker AuNPs were then synthesized by conducting isotropical growth on the ∼15 nm thick AuNPs in diluted CTAB solution. For isotropical growth, briefly, the growth solution was prepared by mixing 1 mL of 0.25 M HAuCl_4_, 0.055 mL of 0.1 M AA, and water (8 mL) with 1 mL of 0.1 M CTAB solution by order. Then, the isotropical growth reaction was initialized by adding 0.3 mL of the AuNP seed solution (15 nm) into the above freshly prepared isotropical growth solution. After this step, the expected thickness of AuNPs was at least 60 nm. Next, Au@PtNPs and Au@PdNPs were both prepared by this recipe; 250 μL of the AuNP (60 nm) stock solution was first mixed with 60 μL of 20 mM AA. Subsequently, the solution was brought to be mixed by vortexing for 10 s. Next, 15 μL of 10 mM H_2_PtCl_6_ solution was introduced followed by vortexing for 10 s again. The solution was kept at room temperature for 1 h without disturbing until the completion of bimetallic NP growth. For purification, the solution was centrifuged at 800 rcf for 2 min, twice. Finally, after removal of the supernatant, the Au@PtNPs were dissolved in 1.0 mL of water and sonicated for 20 s. In parallel, 30 μL of 10 mM H_2_PtCl_4_ was introduced to the mixture of AuNP (60 nm) stock solution and 60 μL of 20 mM AA following the same purification process and redispersed in 1.0 mL of water for further use.

### Preparation of AgNPs, Ag@PdNPs, and Ag@PtNPs

The triangular AgNPs were prepared via a seed-mediated procedure. First, a suspension of seeds was prepared using 25 mL of 0.1 mM silver nitrate (AgNO_3_), 1.5 mL of 30 mM trisodium citrate (Na_3_CA), and 1.5 mL of 0.7 mM PVP (*M*_W_ = 29,000). Under magnetic stirring, Na_3_CA was added into the AgNO_3_ solution and left at room temperature for 2 h.

An aqueous solution of the above prepared PVP, 60 μL of 30 wt % H_2_O_2_, and 0.25 mL of 100 mM NaBH_4_ were added all at once and kept at room temperature for 0.5 h. Ten milliliters of the as-prepared AgNP seeds was mixed with 10 mL of aqueous solution containing AA (1.2 mM) and PVP (1.12 mM, in terms of the repeating unit). Then, 10 mL of AgNO_3_ (0.6 mM) was added to the mixture using a syringe pump at a rate of 10 mL/h under magnetic stirring. After reaction for 5 min, 10 mL of the reaction solution (30 mL in total) was taken out for use as the seeds for another round of growth. This seeded growth process was repeated eight more times, and the reaction conditions were kept the same for all rounds of growth. The product obtained after each round of growth was collected by centrifugation at 10,000 rpm for 12 min and washed with water three times. Ag@PdNPs and Ag@PtNPs were prepared in a similar manner as Au@PdNPs and Au@PtNPs by galvanic replacement. 250 μL of the AgNP (60 nm) stock solution was first mixed with 40 μL of 20 mM AA. Subsequently, the solution was brought to be mixed by vortexing for 10 s. Next, 12 μL of 8 mM H_2_PtCl_6_ solution was introduced followed by vortexing for another 10 s. The solution was kept at room temperature for 1 h without disturbing until the completion of bimetallic NP growth. For purification, the solution was centrifuged twice for 2 min at 8000 rcf. Finally, after removal of the supernatant, the Ag@PtNPs were dissolved in 1.0 mL of water and sonicated for 20 s. For reassembly, 30 μL of 8 mM of H_2_PtCl_4_ was introduced to the mixture of AgNP (60 nm) stock solution and 40 μL of 20 mM AA following the same purification process and redispersed in 1.0 mL of water for further use. AuNPs/AgNPs, Au@PdNPs/Ag@PdNPs, and Au@PtNPs/Ag@PtNPs were then prepared by mixing the corresponding pair in the same tube for further use.

#### Formation of 4-MPM, 4-MBA, and 4-NBT Monolayer on the Mono/Bimetallic Particles

A drop of the as-synthesized nanoparticle stock solution was first deposited on a precleaned Si wafer and incubated for 30 min. Subsequently, the NP-deposited Si wafer was immersed in 2 mM ethanolic solution of 4**-**MPM, 4-MBA, or 4-NBT solution for 1 h to form a monolayer of 4-MPM, 4-MBA, and 4-NBT on different types of particles. Finally, the modified sample was sonicated in ethanol for 3 min for removal of the uncoordinated 4-MPM, 4-MBA, and 4-NBT molecules.

#### TERS Probe Fabrication

Silicon AFM probes with related parameters, force constant 2.7 N/m and resonance frequency 50–80 kHz, were purchased from Appnano (Mountain View, CA). Then, metal evaporation was carried out for coating the AFM tips with a layer of gold. Briefly, 2 of the probes were fixed onto each of the clamped device, and 10 of the devices were put in the thermal evaporator chamber (MBrown, Stratham, NH). During metal deposition, the pressure was kept at ∼1 × 10^–6^ mbar. Then, gold pellets (Kurt J. Lesker, Efferson Hills, PA) were thermally evaporated at a constant 0.2 Å·s^–1^ rate. After 70 nm of Au was deposited on the AFM tips, the evaporation was stopped and cooled down to room temperature. The temperature at the tip surface and deposition chamber was ∼50 °C.

#### TERS Measurement

AFM-TERS and AFM scanning were carried out on the AIST-NT-HORIBA system equipped with a 632.8 nm continuous wavelength laser. Laser light was brought to the sample surface in a side-illumination geometry with a 100× Mitutoyo microscope objective. The scattering electromagnetic radiation was also collected with the same objective and directly introduced to a fiber-coupled Horiba iHR550 spectrograph equipped with a Synapse EM-CCD camera (Horiba, Edison, NJ).

### Quantum Chemical Simulations

Quantum chemical simulations were conducted to investigate the chemical effects on the reactivity of gold and silver nanostructures in the plasmon catalysis reduction of 4-NBT to DMAB. Thereby, we follow our lately introduced computational protocol to address spectroscopic properties as well as the reactivity of plasmonic hybrid systems.^148−151,57^ Accordingly, geometry optimizations for the singlet ground states of 4-NBT and DMAB surface-immobilized on Ag and Au slabs were conducted at the density functional level of theory (DFT). These calculations were based on the projector-augmented wave (PAW) method utilizing the optB88-vdW functional^48^ in a real-space grid of 0.2 Å resolution, implemented in the GPAW program package^49,50^ in cooperation with the ASE interface.^51^ Ag and Au slabs are represented by a 4 × 4 × 3 fcc(111) cluster, resulting in 3 layers each of 16 atoms using an optimized lattice constant of 4.188 and 4.178 Å, respectively. Through the sulfur atom of the thiol moiety, the 4-NBT and DMAB molecules strongly attach to the metal slabs. The subsequent partial structural relaxation was performed employing two-dimensional periodic boundary conditions (*x*- and *y*-direction), while the second and third layers of the metal slabs were frozen to reduce computational costs.

In the case of 4-NBT, the hybrid systems can adopt different configurations. While chemisorption occurs in all configurations due to the sulfur–silver interaction, the orientation of the aromatic moiety with respect to the metal surface varies, leading to differences in physisorption. Moreover, we anticipate a significant influence of surface coverage on the molecular orientation. At high surface coverage, where strong chemical interactions arise from the sulfur–metal bond (i), along with weaker substrate–metal interactions through physisorption (ii), and dispersive interactions between neighboring substrate molecules (iii), configurations with a perpendicular orientation of the aromatic planes relative to the metal surface are favored. Therefore, our simulations for 4-NBT were focused on an orientation in which the plane of the phenyl ring is perpendicular to the metal surface and the hydrogen atom of ^1^C interacts with it. To further examine the dispersive intermolecular interactions among neighboring substrate molecules, we assessed two 4-NBT with parallel orientation. As the dimerization, yielding DMAB, is restricted to neighboring molecules, the structure of DMAB features a more pronounced degree of rigidity, for example, the strong silver–sulfur bond of the monomers allows exclusively the formation of the cis-isomer. Therefore, only the *cis*-isomer orientation was computationally investigated for DMAB.

Subsequently, nonperiodic DFT and time-dependent DFT (TDDFT) simulations were performed using Gaussian 16 program.^52^ A vibrational analysis was carried out for each hybrid system at the CAM-B3LYP/def2-tzvp level^53,54^ of theory (local)^55^ minima on the respective 3*N*-6 dimensional potential energy surface, while the metal cluster was frozen.

Furthermore, excited-state properties, such as excitation energies, transition dipole moments, and electronic characters, were obtained for the 600 lowest singlet excited states at the TDDFT level of theory for each hybrid system. Therefore, the CAM-B3LYP XC functional was applied as for the preliminary ground-state calculations, while the basis set was reduced to def2-svp. It was possible to investigate the electronic nature of light-driven processes in resonance upon 633 nm photoexcitation (1.96 eV) of plasmonic hybrid systems in this manner. In particular, the photoinduced redox chemistry between the metal cluster (Au or Ag) and the respective substrates (4-NBT and DMAB) was assessed. Such a computational approach allows an adequate description of excited-state properties of azobenzenes, as shown lately in comparison with high-level multiconfigurational methods, that is, with respect to excitation energies, excited-state gradients, and resonance Raman intensities. Electronic characters, that is, local excitation of the substrate as well as charge-transfer excitation between the substrate and the metal cluster, were evaluated based on charge density differences (CDDs) (see Tables S1–S3).

### Electrodynamic Simulations

Electrodynamic simulations are performed using the Ansys-Lumerical Multiphysics Suite which makes use of the finite-difference time-domain (FDTD) method to solve Maxwell’s curl equations.^9^ Our model uses a finite silicon (Si) tip coated with 70 nm of gold (Au) with the tip situated on top of a semi-infinite Si substrate. The silver-based triangular nanoplates (AgNPs, Ag@PtNPs, and Ag@PdNPs) are modeled with lateral dimensions of 500 nm and height of 70 nm, while their gold counter parts (AuNPs, Au@PtNPs, and Au@PdNPs) have lateral dimensions of 500 nm and a height of 70 nm. The Pt and Pd coatings are modeled as a 1 nm thick layer around the AuNPs and AgNPs. The tip is placed 0.5 nm above the NP. A plane wave source is incident at 45° from the vertical axis of the tip (*z*-axis), at a wavelength of 633 nm, and with a polarization along the vertical direction. A mesh size of 1 × 1 × 1 nm is used to discretize the tip–substrate region, and a refined mesh size of 0.1 × 0.1 × 0.1 nm is used within the gap region. Perfectly matched layers are used as boundary conditions. The dielectric permittivities tabulated by Palik are used for Si, Au, Pd, and Pt,^10^ and the surrounding medium is set as vacuum. The computational model is shown in Figure S1. The local electric enhancements shown in Figure S1, |**E**/**E**_**0**_|, where **E** is the total electric field and **E**_**0**_ is the incident field from the optical excitation, are calculated and mapped with a 0.1 nm spatial resolution.

## Results and Discussion

### Plasmon-Driven Reduction of 4-NBT to DMAB on AuNPs and AgNPs

**Figure 1:**
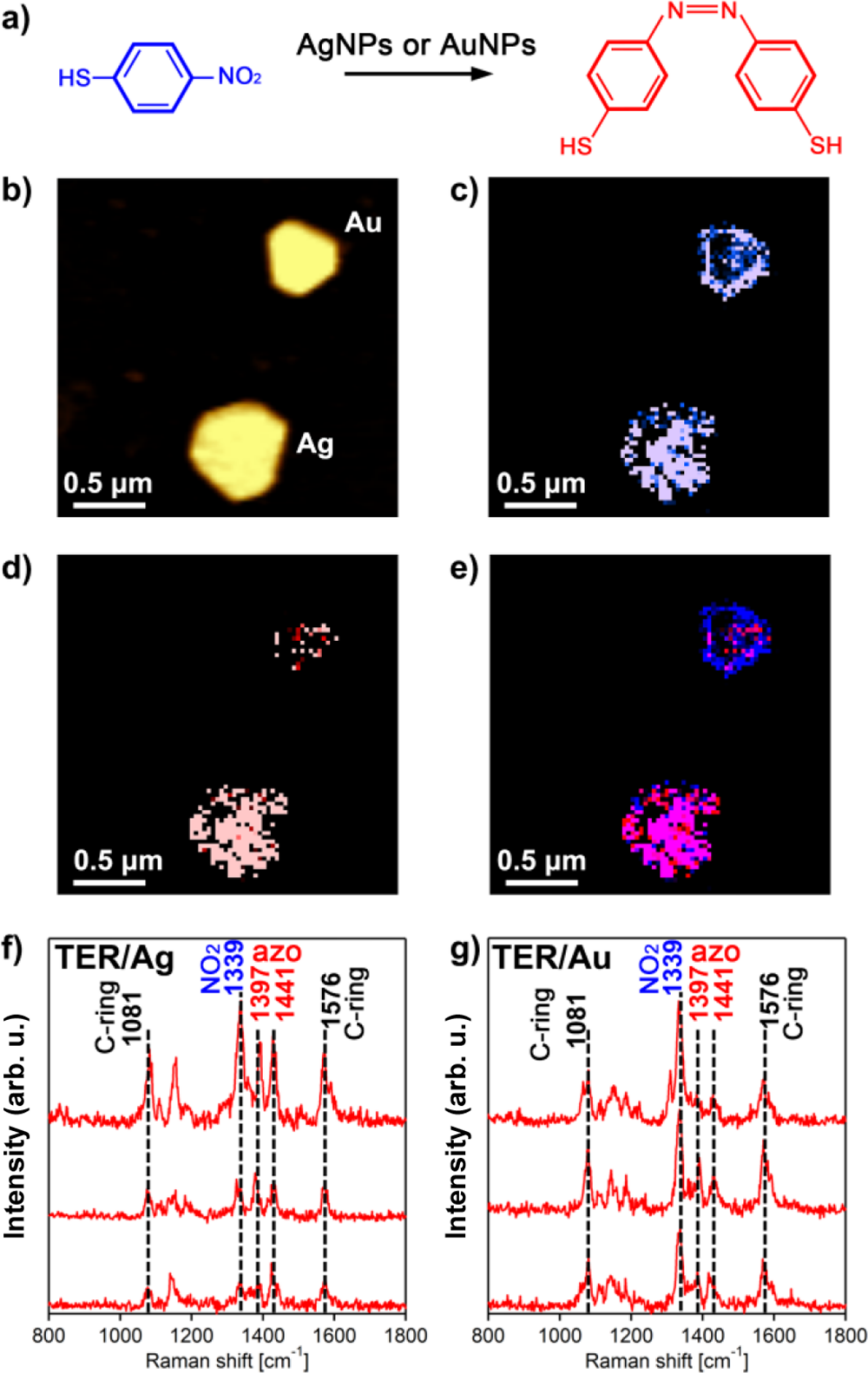
Plasmon-driven transformations of 4NBT into DMAB on AuNPs and AgNPs. (a) Reaction scheme of 4-NBT reduction to DMAB and (b) corresponding AFM image of AuNPs and AgNPs. TERS map of (c) 4-NTP and (d) DMAB, as well as the overlapping TERS image of 4-NTP and DMAB (e) (10 nm per pixel). Intensity of 1339 cm^–1^ band (NO_2_ vibration) of 4-NTP is shown in blue, and intensities of 1397, 1441 cm^–1^ (azo vibration) of DMAB are shown in red. Typical TERS spectra extracted from chemical maps on (f) AgNPs and (g) AuNPs showing the presence of 4-NTP (blue) and DMAB (red). Scale bar is 500 nm in each map. The intensity ranges of 4-NTP, 4-ATP, and DMAB are 10^4^–10^5^.

We first compared the plasmon reactivity of AuNPs and AgNPs in the photocatalytic reduction of 4-NBT into DMAB (Figure 1a). To this aim, a monolayer of 4-NBT was formed on their surfaces by incubation of a silicon wafer with both AuNPs and AgNPs present on it in an ethanolic solution of 4-NBT (Figure 1b–e). This molecular analyte has a distinct vibrational spectrum with only three major vibrational bands centered at 1081, 1339, and 1576 cm^–1^ (Figure 1f,g). Evidence of DMAB formation can be found by the appearance of two bands at 1397 and 1441 cm^–1^, which both can be associated with its N=N moiety^18,31^ (Figure 1f,g).

We observed the plasmon-driven reduction of 4-NBT into DMAB on both AuNPs and AgNPs. However, AgNPs exhibited yields of DMAB much greater than AuNPs. We also found DMAB signals over the entire surface of AgNPs rather than merely at some specific surface sites, such as corners and edges (Figure 1d,e).

**Figure 2:**
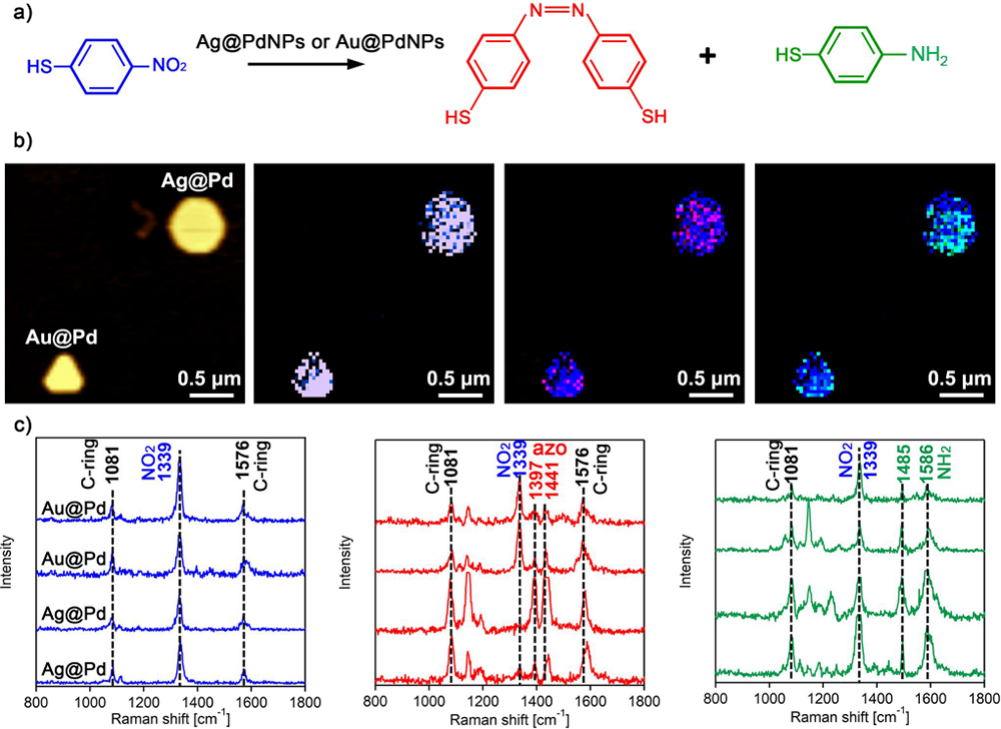
Plasmon-driven transformations of 4NBT into DMAB and 4-ATP on Au@PdNPs and Ag@PdNPs. (a) Reaction scheme of 4-NBT reduction to DMAB and 4-ATP. (b) Corresponding AFM image of AuNPs and AgNPs with a TERS map of 4-NTP, as well as the overlapping TERS image of 4-NTP and DMAB and 4-NBT and 4-ATP (10 nm per pixel). Intensity of 1339 cm^–1^ band (NO_2_ vibration) of 4-NTP is shown in blue, intensities of 1397 and 1441 cm^–1^ (azo vibration) of DMAB are shown in red, and intensity of 1586 cm^–1^ (NH_2_ vibration) of 4-ATP is shown in green. Typical TERS spectra (c) of 4-NBT, DMAB, and 4-ATP extracted from chemical maps of Au@PdNPs and Ag@PdNPs. Scale bar is 500 nm in each map. The intensity range of 4-NTP, 4-ATP, and DMAB are 10^4^–10^5^.

Next, we analyzed the plasmonic properties of Au@PdNPs and their silver analogues, Ag@PdNPs. As discovered by Li and co-workers, on Au@PdNPs, the reduction of 4-NBT yielded not only DMAB, as on their monometallic analogues, but also 4-ATP (Figure 2a). The presence of this molecule could be witnessed by two vibrational bands at 1485 and 1586 cm^–1^ that were observed neither in 4-NBT nor in DMAB.^18,31^ TERS imaging revealed that both Au@PdNPs and Ag@PdNPs were able to reduce 4-NBT to both DMAB and 4-ATP (Figure 2b,c). At the same time, we observed significantly larger yields of both DMAB and 4-ATP on Ag@PdNPs than on Au@PdNPs. We also found that on Au@PdNPs, 4-ATP is primarily formed along the perimeter of the nanoplates, whereas such site-specific localization of 4-ATP formation was not observed on Ag@PdNPs. Neither on Ag@PdNPs nor on Au@PdNPs we observed substantial overlap between the surface sites that yielded DMAB and 4-ATP, which is in a good agreement with the previously reported results by Li and co-workers.^31,56^

**Figure 3:**
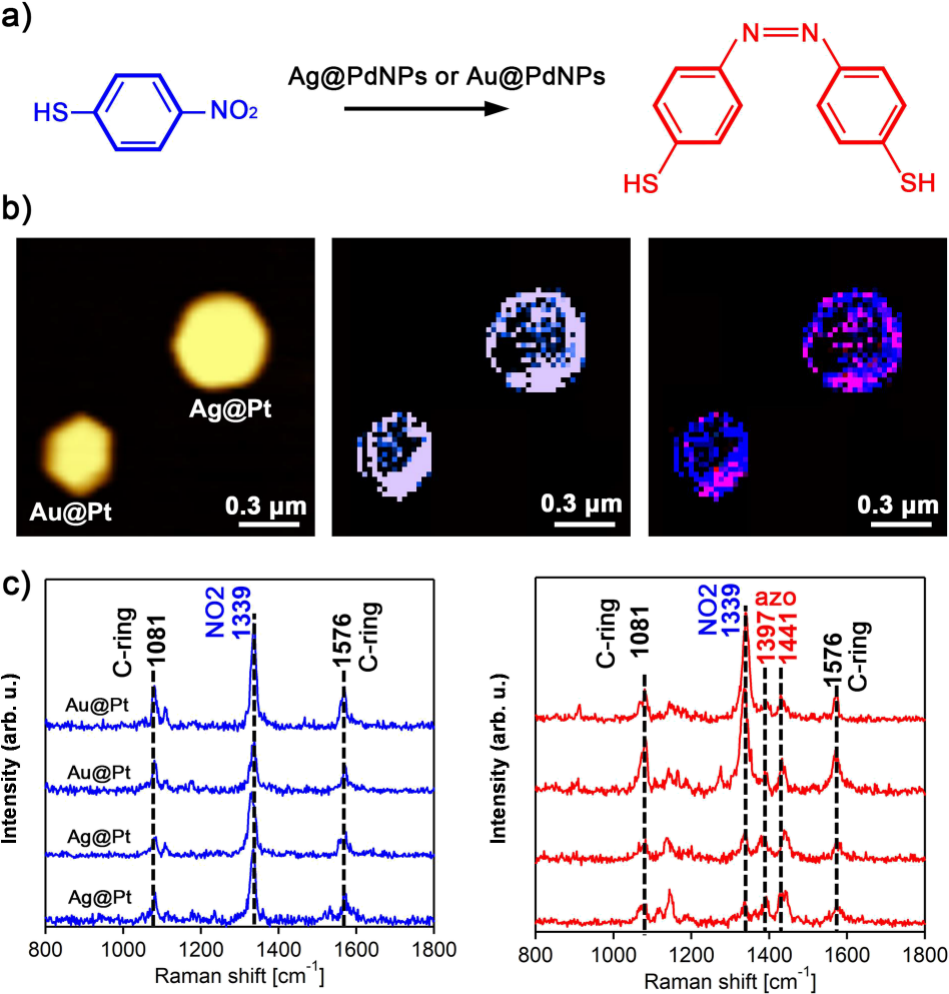
Plasmon-driven transformations of 4NBT into DMAB on Au@PtNPs and Ag@PtNPs. (a) Reaction scheme of 4-NBT reduction to DMAB and (b) corresponding AFM image of AuNPs and AgNPs with TERS map of 4-NTP and the overlapping TERS image of 4-NTP and DMAB (10 nm per pixel). Intensity of 1339 cm^–1^ band (NO_2_ vibration) of 4-NTP is shown in blue, and intensities of 1397 and 1441 cm^–1^ (azo vibration) of DMAB are shown in red. Typical TERS spectra (c) extracted from chemical maps on Ag@PtNPs and Au@PtNPs showing the presence of 4-NTP (blue) and DMAB (red). Scale bar = 300 nm in each map. The intensity range of 4-NTP, 4-ATP, and DMAB are 10^4^–10^5^.

TERS analysis of the photocatalytic performance of Au@PtNPs and Ag@PtNPs demonstrated that these bimetallic nanostructures, like their monometallic analogues, were capable of reducing 4-NBT only to DMAB (Figure 3). Similar to AuNPs and AgNPs, we observed a much greater yield of DMAB on Ag@PtNPs in comparison to Au@PtNPs. We also found that the formation of DMAB on Au@PtNPs was taking place primarily at the perimeter of the nanostructures, whereas on Ag@PtNPs, both the perimeter and central part of the nanostructures exhibited strong catalytic reactivity.

**Figure 4:**
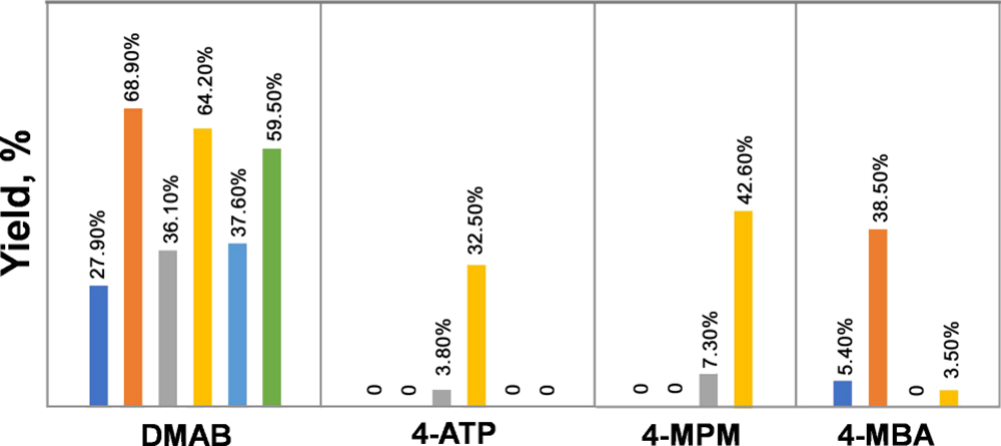
Histogram of the yield of DMAB and 4-ATP on AuNPs (dark blue), AgNPs (red), Au@PdNPs (gray), Ag@PdNPs (yellow), Au@PtNPs (light blue), and Ag@PtNPs (green).

Our results showed that AgNPs exhibit an enhanced reactivity in the investigated plasmon-driven reactions compared to AuNPs. Specifically, we observed ∼2.5 times higher yields of DMAB on AgNPs compared to their gold analogues (Figure 4). A similar relationship between the antenna metal and the DMAB yield was observed for bimetallic NPs. Specifically, we observed ∼2 times higher yields of DMAB on Ag@PdNPs and Ag@PtNPs compared to their gold-based analogues, Au@PdNPs and Au@PtNPs, respectively. We also found that Ag@PdNPs demonstrated yields of 4-ATP nearly 10 times greater compared to those of Au@PdNPs (Figure 4). It should be noted that this reaction product was not observed on any of the other bimetallic or monometallic nanostructures.

**Figure 5:**
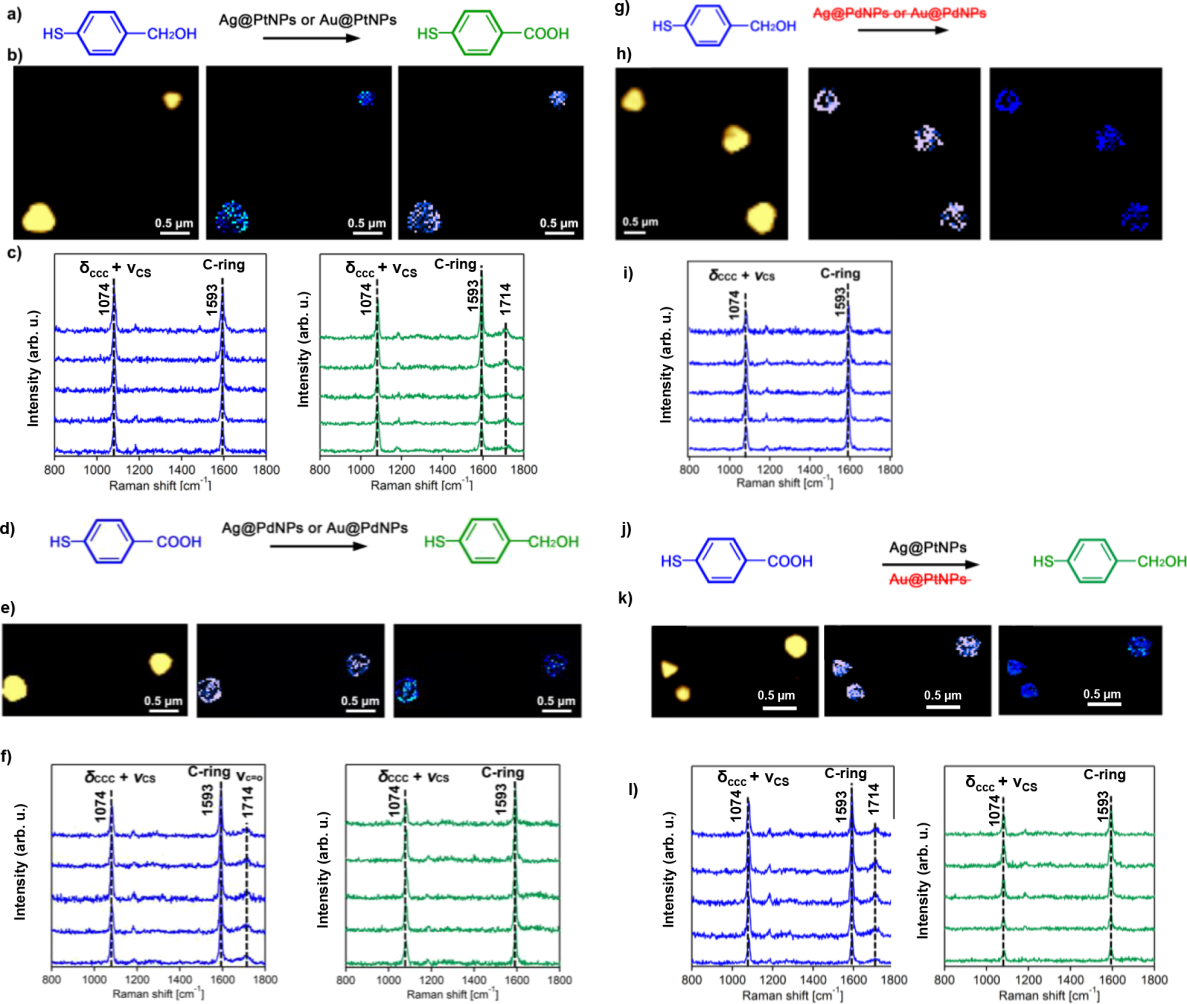
Plasmon-driven redox reactions between 4-MPM and 4-BMA on Au@PtNPs, Ag@PtNPs, Au@PdNPs, and Ag@PdNPs. (a–j) Reaction scheme of redox reactions between 4-MPM and 4-MBA. (b, e, h, and k) Corresponding AFM images of bimetallic nanostructures with TERS maps of 4-MPM (blue) and overlapping 4-MPM and 4-MBA (green) (10 nm per pixel). Intensity of 1074 cm^–1^ band (δ(CCC) + ν(CS)) of 4-MPM is shown in blue, and intensities of 1714 cm^–1^ (COOH) of 4-MBA are shown in green. Typical TERS spectra (c, f, i, and l) extracted from chemical maps of bimetallic nanostructures showing the presence of 4-MPM (blue) and 4-MBA (green). Scale bar is 500 nm in each map.

As previously demonstrated by Li and Kurouski, Au@PtNPs were capable of plasmon-driven oxidation of 4-MPM into 4-MBA.^33^ Expanding upon this, we investigated the yields of 4-MBA on Au@PtNPs and Ag@PtNPs (Figure 5). We found that both Au@PtNPs and Ag@PtNPs were able to perform plasmon-driven oxidation of 4-MPM to 4-MBA. However, we observed substantially greater yields of 4-MBA on Ag@PtNPs than on Au@PtNPs. We also found that the reversed reaction, that is, reduction of 4-MAB to 4-MPM, could be catalyzed by both Au@PdNPs and Ag@PdNPs (Figure 4). Similar to their Pt analogues, Ag@PdNPs demonstrated an enhanced efficiency in plasmon-driven reduction of 4-MBA than in the case of Au@PdNPs. It is important to note that both Au@PdNPs and Ag@PdNPs demonstrated exclusive reductive properties. Therefore, 4-MPM related signals were not observed on their surfaces. At the same time, Ag@PtNPs were able to reduce 4-MBA to 4-MPM, whereas such a reaction was not evident on their gold analogues, Au@PtNPs (Figure 5).

In summary, we can conclude that Ag@PdNPs demonstrated ∼6 times greater reduction properties in 4-MBA to 4-MPM conversion to Au@PdNPs (Figure 4). Furthermore, an ∼8 times greater efficiency in the plasmon-driven reduction of 4-MPM to 4-MBA oxidation compared to that of Au@PtNPs was determined for Ag@PtNPs. Finally, only a slightly greater yield of 4-MBM to 4-MBA oxidation was observed on Ag@PdNPs compared to Au@PdNPs (Figure 4).

**Figure 6:**
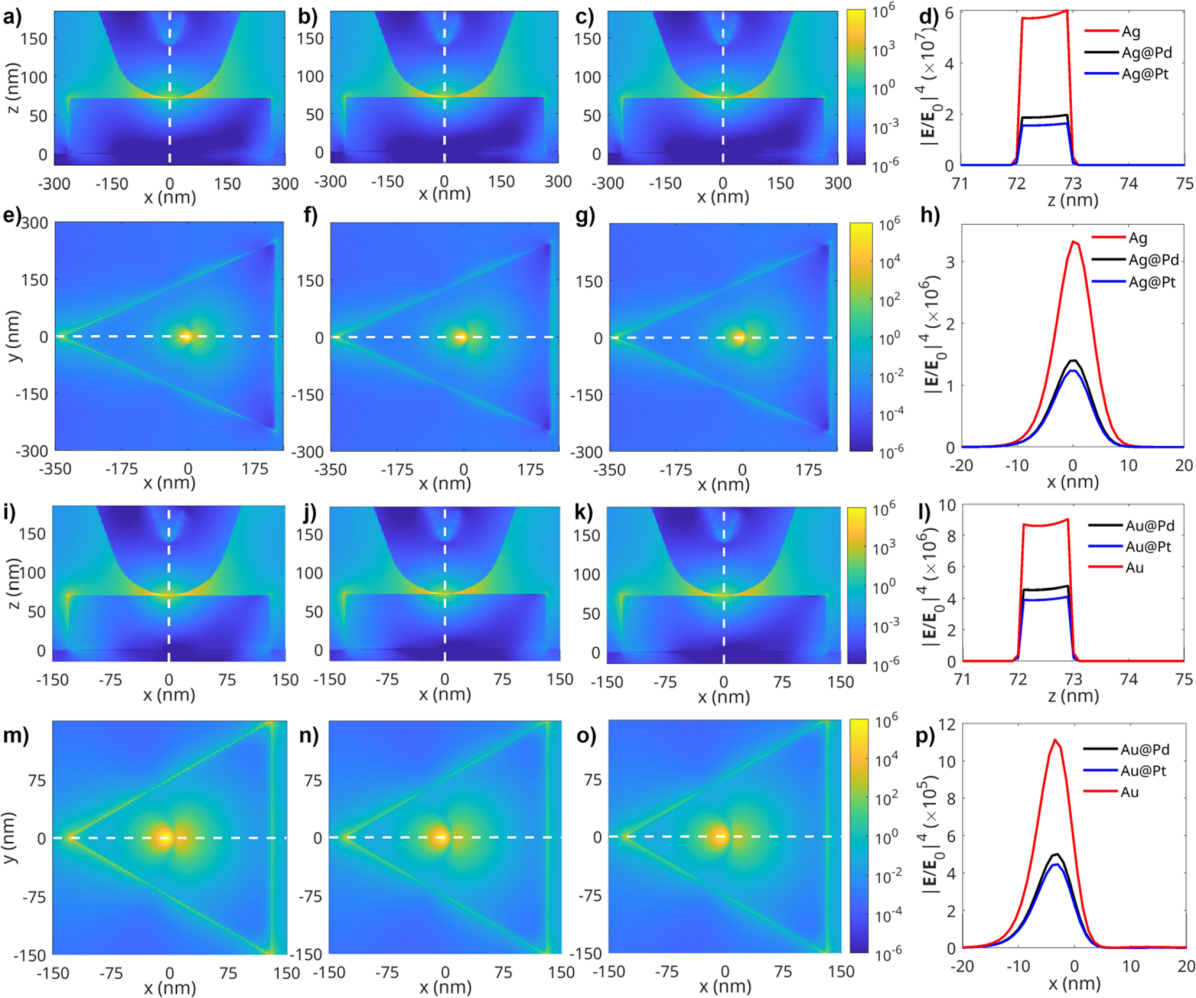
Spatial distributions of the plasmonic TERS enhancement factor, |**E**/**E**_**0**_|^4^, calculated for AgNPs, Ag@PdNPs, Ag@PtNPs, AuNPs, Au@PdNPs, and Au@PtNPs. (a–c) Cross-sectional (*x*,*z*) maps in the tip–substrate region of AgNPs, Ag@PdNPs, and Ag@PtNPs, respectively. (d) Field profiles for the gap region, calculated along the *z*-axis (white dashed lines in panels a–c). (e–g) Cross-sectional (*x*,*y*) maps on the top surfaces of AgNPs, Ag@PdNPs, and Ag@PtNPs, respectively. Each map is calculated at a single excitation wavelength of 633 nm. (h) Field profiles on the NP surface, calculated along the *x*-axis (white dashed lines in panels e–g). (i–k) Cross-sectional (*x*,*z*) maps in the tip–substrate region of AuNPs, Au@PdNPs, and Au@PtNPs, respectively. (l) Field profiles for the gap region, calculated along the *z*-axis (white dashed lines in panels i–k). (m–o) Cross-sectional (*x*,*y*) maps on the top surface of AuNPs, Au@PdNPs, and Au@PtNPs, respectively. Each map is calculated at a single excitation wavelength of 633 nm. (p) Field profiles on the NP surface, calculated along the *x*-axis (white dashed lines in panels m–o).

In order to investigate the underlying physical cause of the experimentally observed superior plasmonic performance of Ag-based compared to Au-based mono- and bimetallic nanoplates in plasmon-driven reactions, we performed FDTD calculations. Our results showed that AgNPs exhibit an ∼8 times higher intensity of the (plasmonic) near-field than AuNPs; see Figure 6.^36^ Similar differences in the intensity of the plasmonic near-field were observed between the corresponding Au- and Ag-bimetallic nanoplates. These results demonstrate that the plasmonic nature of the antenna metal determines the overall reactivity of both monometallic and bimetallic nanostructures.

Catalytic metals deplete the electric field generated by plasmonic nanostructures due to their lossy nature. One may also expect that Pd and Pt can also shift the LSPR of such nanostructures due to the change in the local dielectric environment. Both effects were observed in the electrodynamic simulations resulting in a lower local electric field for the case of bimetallic nanostructures (Figure 6d,h,l,p). Thus, Ag@PtNPs and Ag@PdNPs should exhibit lower yields of reaction products compared to AgNPs. Indeed, we observed a lower yield of DMAB on Ag@PtNPs (59.5%) and Ag@PdNPs (64.2%) compared to the yield of DMAB on AgNPs (68.9%), as seen in Figure 5. However, the opposite relationship was determined for Au@PtNPs, Au@PdNPs, and AuNPs. Specifically, we observed higher yields of DMAB on Au@PtNPs (37.6%) and Au@PdNPs (36.1%) compared to AuNPs (27.9%). These results suggest that other factors, such as interplay between plasmonic and catalytic metals, in addition to the electric field could play a role in the ultimate plasmonic performance of bimetallic nanostructures.

Our investigation encompassed not only the impact of electromagnetic effects on diverse behaviors observed from Ag and Au nanostructures in plasmon-driven reactions but also the impact of the underlying electronic structure on the ground and excited states by means of quantum chemical simulations. To this aim, we employed periodic DFT and nonperiodic TDDFT simulations to model the surface-immobilized 4-NBT on Ag vs Au slabs based on the 600 lowest-energy excited states of the molecular hybrid system models. The inclusion of such a large number of electronic states is essential to account for the electronic transitions accessible within the experimental laser excitation region (1.96 eV, equivalent to 633 nm). Further details regarding the computational setup are available in the Supporting Information as well as in the study by Rodriguez and co-workers.^148−151,57^ In the subsequent discussion, we abstained from discussing specific electronic excitations due to the presence of numerous highly mixed weakly absorbing transitions in the plasmonic hybrid system model, particularly in charge-transfer processes involving the metal slab and surface-immobilized substrate. For a comprehensive overview of the transitions, please refer to the Supporting Information.

**Figure 7:**
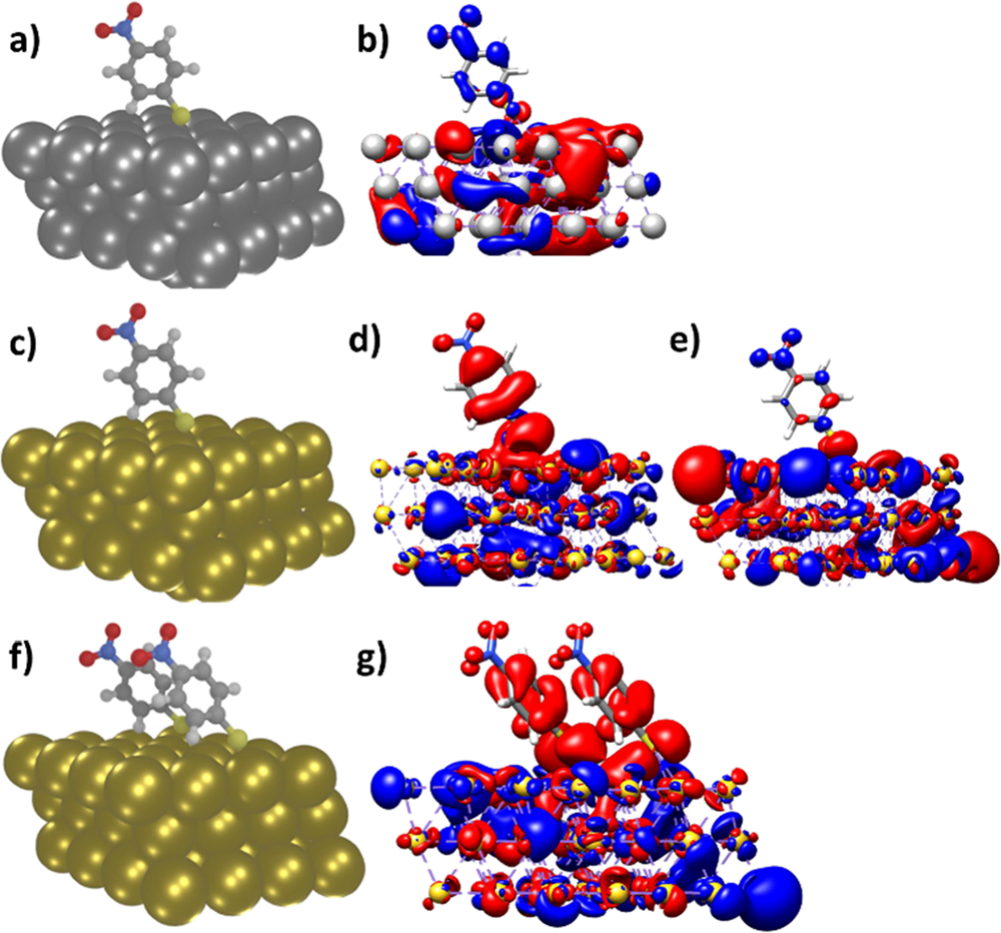
(a) Optimized geometry of 4-NBT on the Ag slab and (b) CDD plots, illustrating metal-to-molecule charge transfer (red to blue). (c) Optimized structure of 4-NBT on the Au slab and (d) CDDs, showcasing molecule-to-metal charge transfer (e) and metal-to-molecule charge transfer (e) (red to blue). (f) Optimized geometry of two parallel 4-NBT molecules on the Au slab and (g) CDDs, demonstrating mainly molecule-to-metal charge transfer (red to blue).

One of the pivotal factors influencing redox reactions in hybrid systems is the direction of charge transfer within the system. In the case of 4-NBT, for the reduction of the nitro group, the transfer of electrons toward this specific group is imperative to enable the sequential progression of the reaction. By analyzing the CDDs of 4-NBT on AgNPs (refer to Figure 7b), it becomes evident that the excited states are predominantly of metal-to-molecule charge-transfer character. In this scenario, charge is transferred from the AgNP toward the 4-NBT molecule, particularly into the π* orbital of the nitro group, which translates to the reduction of 4-NBT. However, the situation differs when AuNPs are involved as a substantial portion of the excited states within the range of laser radiation exhibits the electronic character of molecule-to-metal charge transfer (as illustrated by a specific example in Figure 7d), that is, charge transfer of opposite directionality, while only a minor fraction of the excited states display a metal-to-molecule charge-transfer nature, as exemplified by one such instance in Figure 7e. For a comprehensive understanding of the electronic character of all the excited states, please refer to Tables S1 and S2 in the Supporting Information. These tables provide detailed information regarding the characteristics of the excited states associated with the charge-transfer processes, as discussed. This disparity in electronic characteristics plays a crucial role in the reduction of 4-NBT, and it is one of the key factors contributing to the higher reactivity observed with AgNPs compared to AuNPs.

Additionally, we conducted a comprehensive analysis to investigate the underlying photophysical properties that contribute to the distinct differences in reactivity observed in our experimental results. These differences are characterized by the predominant reactions occurring at the perimeter of AuNPs and robust catalytic reactivity across AgNPs. Notably, DMAB formation was observed in both the perimeter and central regions of the Ag nanostructure, whereas it was primarily restricted to the perimeter of the Au nanoparticles. Our goal was to unravel the fundamental reasons for this variation. To delve into this phenomenon, we conducted a comparative examination of the CDDs for two 4-NBT molecules on the surface of Au nanoparticles (see Figure 7g). Thereby, electronic effects stemming from short-range molecule–molecule interactions with respect to ground- and excited-state properties are taken into account as in the case of high-coverage self-assembled monolayer AuNPs.

Remarkably, our findings demonstrated a substantial increase in the fraction of excited states exhibiting an electronic character of molecule-to-metal charge transfer (associated with 4-NBT oxidation) when considering two neighboring molecules on the Au slab compared to the scenario with only one molecule (see Table S3 in the Supporting Information). This observation presents compelling evidence that in the central part of Au nanostructures, characterized by a dense layer of 4-NBT, the likelihood of the reduction reaction is diminished compared to the edges and corners.

**Figure 8:**
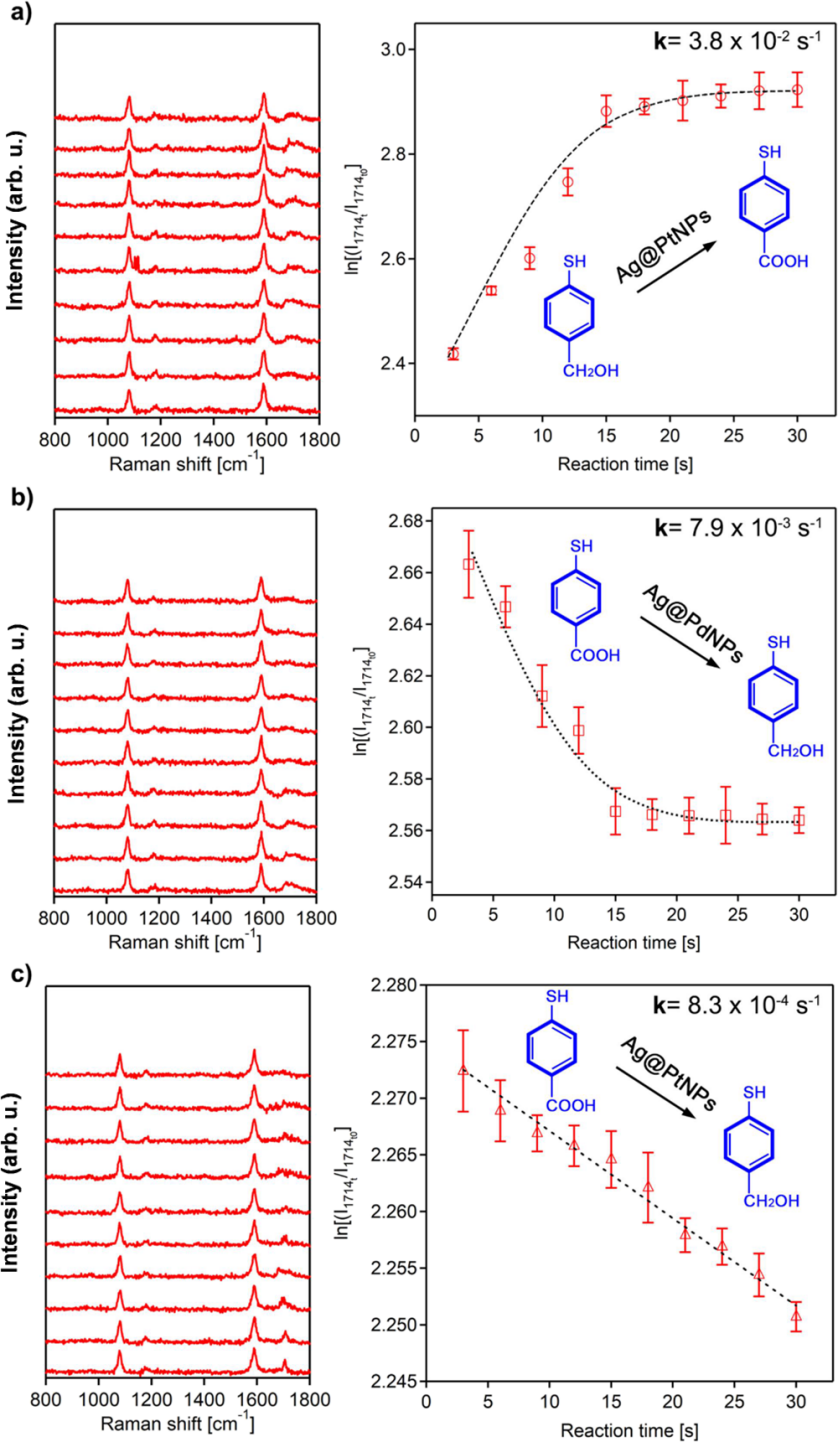
Kinetics of 4-MPM and 4-MBA redox reactions on Ag@PtNPs and Ag@PdNPs. TERS kinetic measurements of (a) 4-MPM to 4-MBA oxidation on Ag@PtNPs, (b) 4-MBA to 4-MPM reduction on Ag@PdNPs, and (c) 4-MBA to 4-MPM reduction on Ag@PtNPs. Rate constants of both oxidation and reduction are based on the intensity ratio of the band at 1714 cm^–1^ (COOH).

Finally, we also used TERS to determine the rates of plasmon-driven transformations discussed above (Figure 8). We acquired a set of TERS spectra from at least three different locations on the surface of Ag@PtNPs. We monitored changes in the intensity of the 1714 cm^–1^ band, which corresponds to the COOH group of 4-MBA, relative to the intensity of 1074 cm^–1^ (δ(CCC) + ν(CS)), as defined in eq 1. This vibrational band remained unchanged for 4-MBA and 4-MPM.
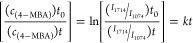
1where *c*_(4-MBA)_*t*_0_ and *c*_(4-MBA)_*t* represent the concentrations of 4-MBA at different reaction times, respectively. *I*_1714_ and *I*_1074_ are the intensities of the bands at 1714 cm^–1^ (MBA) and 1074 cm^–1^ (MPM), respectively. *k* is the rate constant and *t* is the reaction time.^33^

We found that in the case of Ag@PtNPs, the rate of 4-MPM to 4-MBA conversion was *k* = 3.8 × 10^–2^ s^–1^, whereas the rate for the reversed process was 8.3 × 10^–4^ s^–1^ (Figure 8 and Table 1). We also observed that the rate of reduction of 4-MBA to 4-MPM on Ag@PdNPs was 7.9 × 10^–3^ s^–1^. Interestingly, the kinetics of 4-MPM to 4-MBA oxidation on Au@PtNPs and a reversed reduction of 4-MPM to 4-MPM on Au@PdNPs both had a logarithmic shape, whereas the kinetics of 4-MBA reduction on Au@PtNPs had a linear trend (Figure 8).

**Table 1:** Rates of Plasmon-Driven Redox Reactions (s^–1^) between 4-MPM and 4-MBA on Au@PdNPs, Ag@PdNPs, Au@PtNPs, and Ag@PtNPs

	**Au@PdNPs**	**Ag@PdNPs**	**Au@PtNPs**	**Ag@PtNPs**
4-MPM			1.1 × 10^–2^	3.8 × 10^–2^
4-MBA	3.1 × 10^–3^	7.9 × 10^–3^		8.3 × 10^–4^

## Conclusions

**Figure 9:**
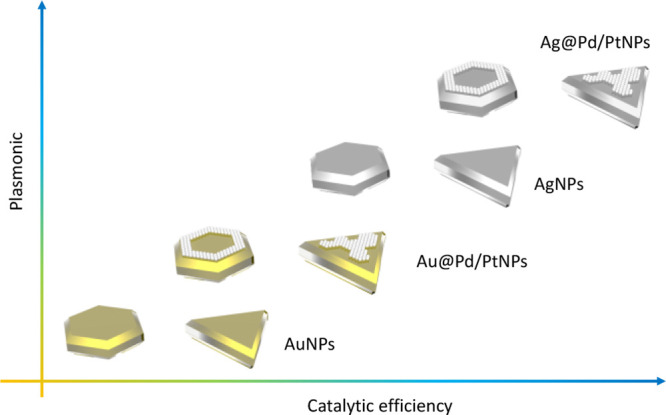
Schematic illustration of plasmonic properties and catalytic efficiency of monometallic and bimetallic Au- and Ag-based NPs.

We showed that AgNPs and silver-based bimetallic nanostructures demonstrate much greater yields and exhibit higher rates of plasmon-driven chemical reactions, as shown in Figure 9. Specifically, we observed 3–10 times enhanced yields and ∼3 times higher rates of plasmon-driven redox reactions on AgNPs and Ag-based bimetallic nanostructures compared to their Au analogues. Our electrodynamic simulations reveal that similar intensity differences of the electromagnetic field are observed between Ag-based and Au-based mono- and bimetallic nanostructures. Therefore, we conclude that the rate and yield of plasmon-driven processes have a strong correlation with the nature of the plasmonic antenna in such catalytic systems. These findings open new synthetic strategies that can be utilized to improve the plasmonic reactivity of mono- and bimetallic nanostructures.

## Supporting Information Available

3D projection of the computational FDTD TERS model and charge density differences in DFT calculations (PDF)
