# Supplementary material for: Role of Plasmonic Antenna in Hot Carrier-Driven Reactions on Bimetallic Nanostructures
Source: J Phys Chem C Nanomater Interfaces. 2023 Nov 9;127(46):22635–45. doi: 10.1021/acs.jpcc.3c06520 (PMC10863061; doi:10.1021/acs.jpcc.3c06520)
Supplement: Supplementary file 1 — jp3c06520_si_001.pdf [file jp3c06520_si_001.pdf]

The Role of Plasmonic Antenna in Hot-Carrier-Driven Reactions on Bimetallic Nanostructures

Zhandong Li,[1] Joel Rigor,[2] Sadaf Ehtesabi,[3] Siddhi Gojare,[3] Stephan Kupfer,[3] Stefanie Gräfe,[3] Nicolas Large[2] and Dmitry Kurouski[1,4]*

1. Department of Biochemistry and Biophysics, Texas A&M University, College Station, Texas 77843, United States
2. Department of Physics and Astronomy, The University of Texas at San Antonio, Texas 78249, United States.
3. Institute of Physical Chemistry and Abbe Center of Photonics, Friedrich Schiller University Jena, Helmholtzweg 4, 07743 Jena, Germany.
4. The Institute for Quantum Science and Engineering, Texas A&M University, College Station, Texas, 77843, United States

Email: dkurouski@tamu.edu

Supporting Information

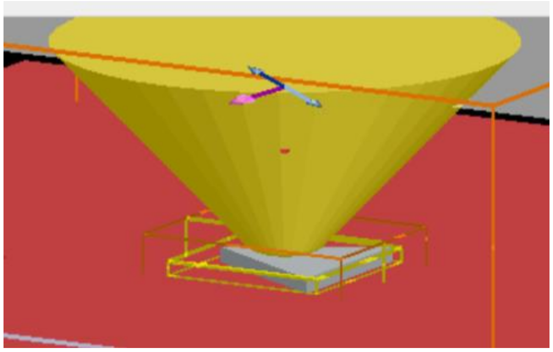

Figure S1. Computational TERS Model. We model the TERS system with a finite silicon (Si) tip coated with 70 nm of gold (Au) located 0.5 nm above a triangular Au nanoplate of 70 nm in height and 300 nm in lateral dimensions with a 1 nm layer of Pt or Pd. The optical source is defined by its wavevector (purple arrow) and linear polarization (blue arrows).

Table S1: Charge density differences (CDDs) illustrating the nature of the low-lying dipole-allowed excitations of 4-NBT on Au surface. Charge transfer takes place from red to blue.

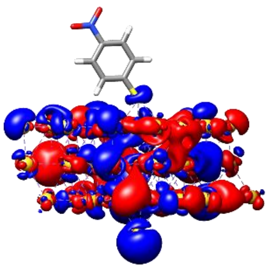<br>State 37<br>Energy: 1.392 eV<br>Osc.: 0.011	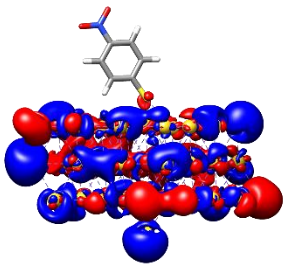<br>State 44<br>Energy: 1.575 eV<br>Osc.: 0.017	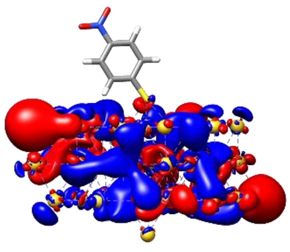<br>State 52<br>Energy: 1.720 eV<br>Osc.: 0.021	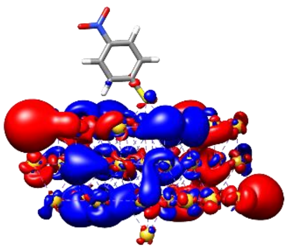<br>State 53<br>Energy: 1.751 eV<br>Osc.: 0.015
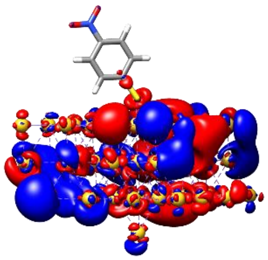<br>State 60<br>Energy: 1.867 eV<br>Osc.: 0.010	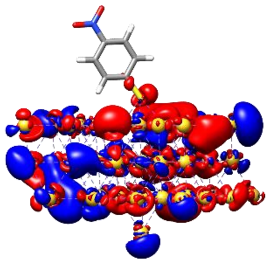<br>State 63<br>Energy: 1.912 eV<br>Osc.: 0.044	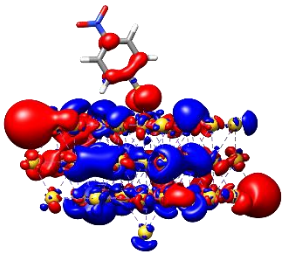<br>State 73<br>Energy: 2.044 eV<br>Osc.: 0.011	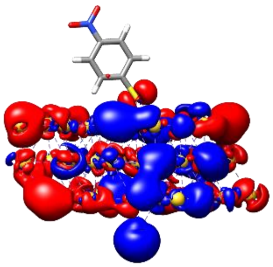<br>State 82<br>Energy: 2.156 eV<br>Osc.: 0.010
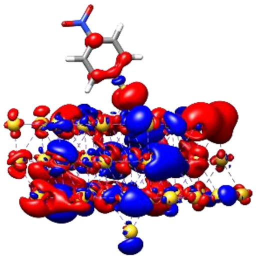<br>State 84<br>Energy: 2.174 eV<br>Osc.: 0.045	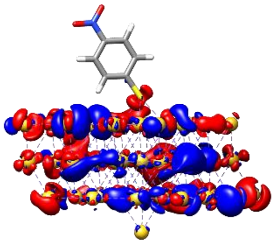<br>State 86<br>Energy: 2.219 eV<br>Osc.: 0.010	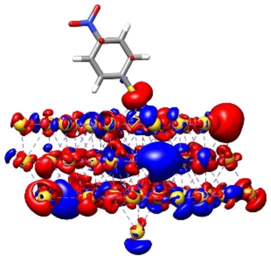<br>State 88<br>Energy: 2.229 eV<br>Osc.: 0.047	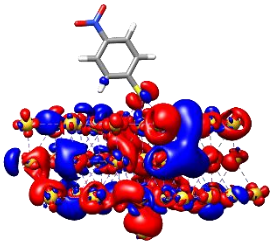<br>State 89<br>Energy: 2.258 eV<br>Osc.: 0.011

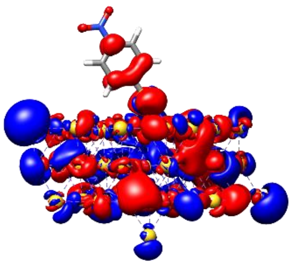<br>State 90<br>Energy: 2.288 eV<br>Osc.: 0.012	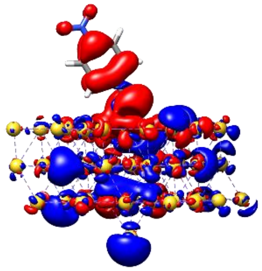<br>State 93<br>Energy: 2.311 eV<br>Osc.: 0.017	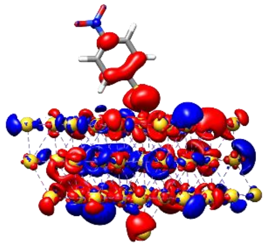<br>State 97<br>Energy: 2.376 eV<br>Osc.: 0.035	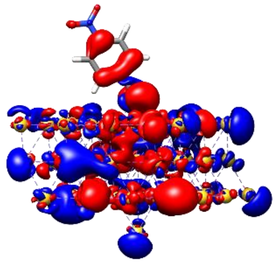<br>State 99<br>Energy: 2.390 eV<br>Osc.: 0.011
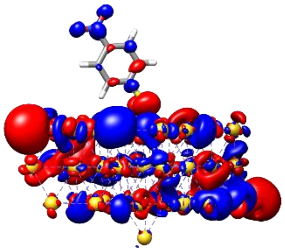<br>State 100<br>Energy: 2.409 eV<br>Osc.: 0.030	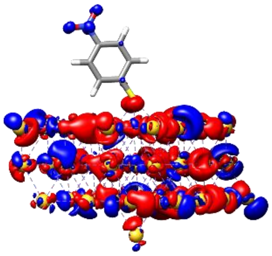<br>State 101<br>Energy: 2.414 eV<br>Osc.: 0.036	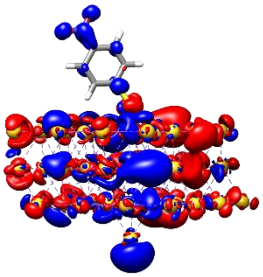<br>State 103<br>Energy: 2.449 eV<br>Osc.: 0.024	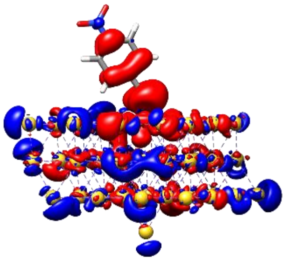<br>State 104<br>Energy: 2.457 eV<br>Osc.: 0.046
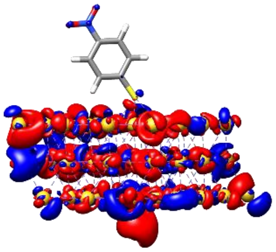<br>State 105<br>Energy: 2.459 eV<br>Osc.: 0.026	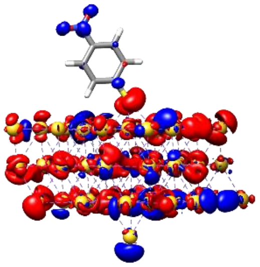<br>State 106<br>Energy: 2.475 eV<br>Osc.: 0.025	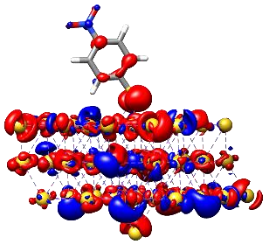<br>State 109<br>Energy: 2.510 eV<br>Osc.: 0.010	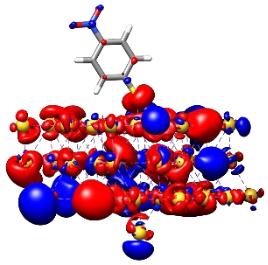<br>State 111<br>Energy: 2.523 eV<br>Osc.: 0.023
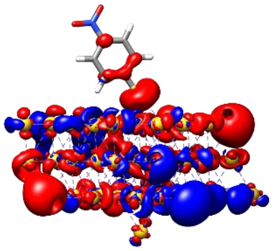<br>State 112<br>Energy: 2.526 eV<br>Osc.: 0.019	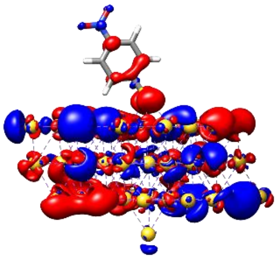<br>State 115<br>Energy: 2.563 eV<br>Osc.: 0.012	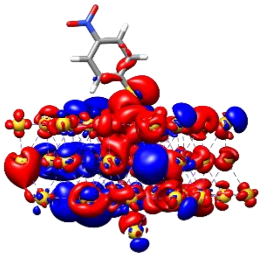<br>State 117<br>Energy: 2.591 eV<br>Osc.: 0.010	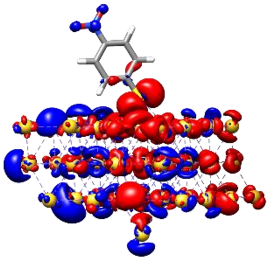<br>State 119<br>Energy: 2.602 eV<br>Osc.: 0.089

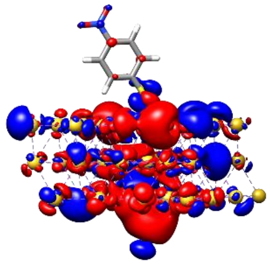<br>State 121<br>Energy: 2.634 eV<br>Osc.: 0.045	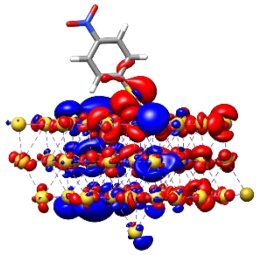<br>State 123<br>Energy: 2.646 eV<br>Osc.: 0.091	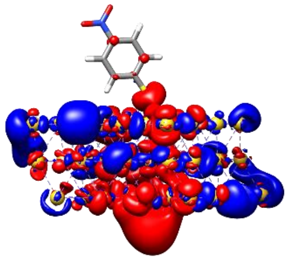<br>State 124<br>Energy: 2.658 eV<br>Osc.: 0.014	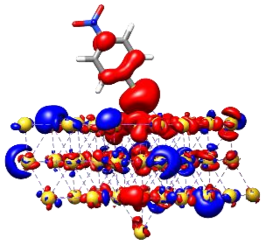<br>State 126<br>Energy: 2.673 eV<br>Osc.: 0.081
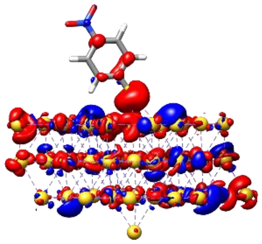<br>State 127<br>Energy: 2.694 eV<br>Osc.: 0.086	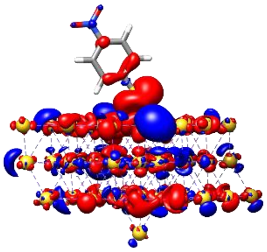<br>State 131<br>Energy: 2.718 eV<br>Osc.: 0.045	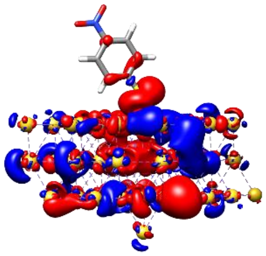<br>State 132<br>Energy: 2.746 eV<br>Osc.: 0.077	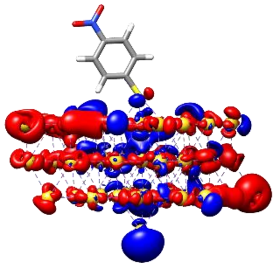<br>State 133<br>Energy: 2.754 eV<br>Osc.: 0.097
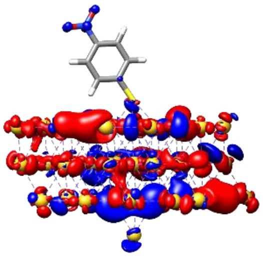<br>State 135<br>Energy: 2.772 eV<br>Osc.: 0.017	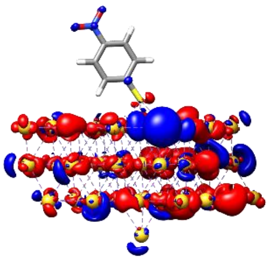<br>State 136<br>Energy: 2.780 eV<br>Osc.: 0.043	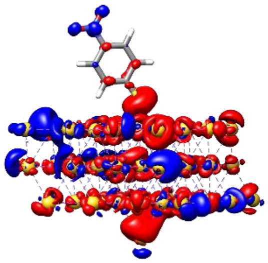<br>State 138<br>Energy: 2.796 eV<br>Osc.: 0.010	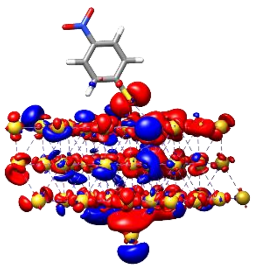<br>State 139<br>Energy: 2.805 eV<br>Osc.: 0.033
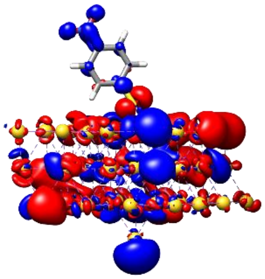<br>State 140<br>Energy: 2.813 eV<br>Osc.: 0.038	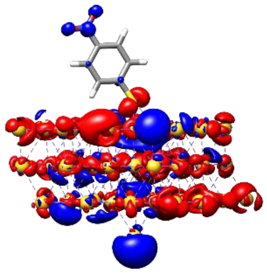<br>State 144<br>Energy: 2.844 eV<br>Osc.: 0.010	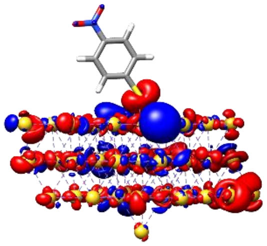<br>State 145<br>Energy: 2.848 eV<br>Osc.: 0.070	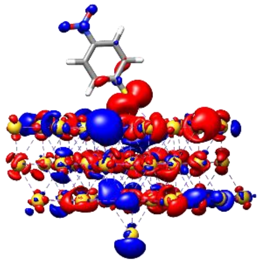<br>State 146<br>Energy: 2.863 eV<br>Osc.: 0.020

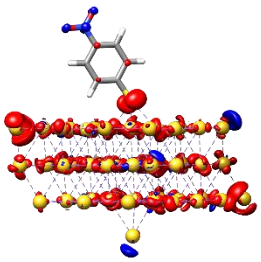<br>State 147<br>Energy: 2.875 eV<br>Osc.: 0.049	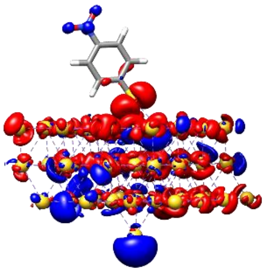<br>State 148<br>Energy: 2.885 eV<br>Osc.: 0.071	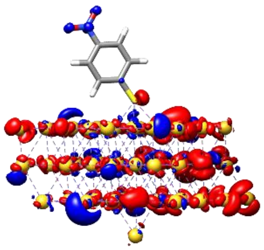<br>State 151<br>Energy: 2.903 eV<br>Osc.: 0.106	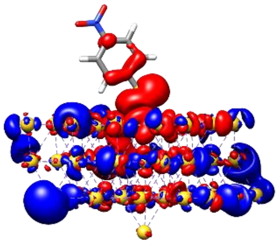<br>State 152<br>Energy: 2.921 eV<br>Osc.: 0.038
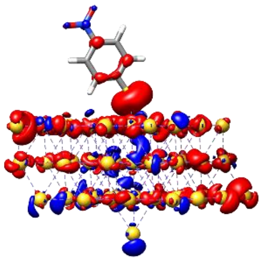<br>State 153<br>Energy: 2.926 eV<br>Osc.: 0.048	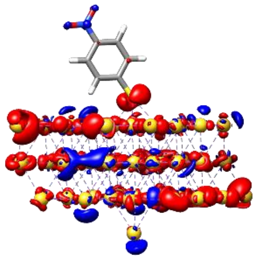<br>State 154<br>Energy: 2.933 eV<br>Osc.: 0.028	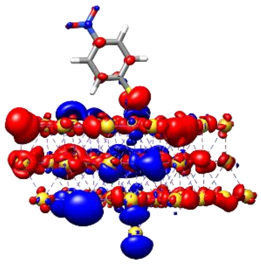<br>State 155<br>Energy: 2.943 eV<br>Osc.: 0.017	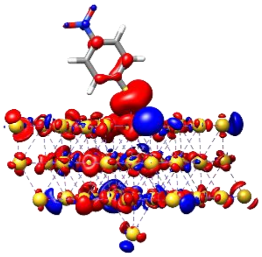<br>State 156<br>Energy: 2.946 eV<br>Osc.: 0.067
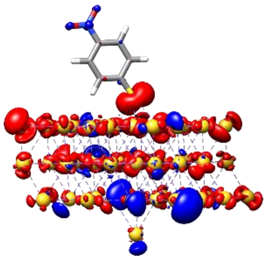<br>State 158<br>Energy: 2.961 eV<br>Osc.: 0.067	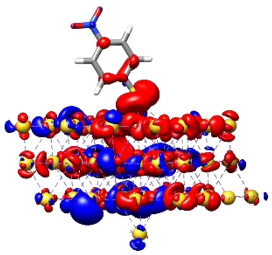<br>State 159<br>Energy: 2.967 eV<br>Osc.: 0.035		

Table S2: Charge density differences (CDDs) illustrating the nature of the low-lying dipole-allowed excitations of 4-NBT on Ag surface. Charge transfer takes place from red to blue.

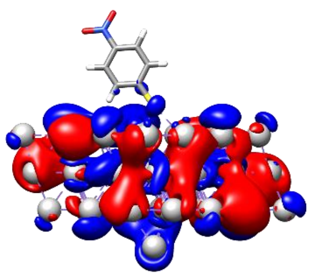<br>State 44<br>Energy: 1.491 eV<br>Osc.: 0.014	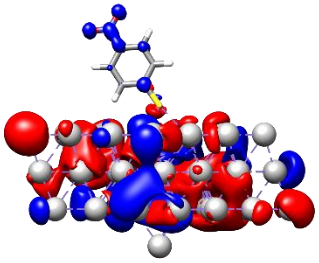<br>State 51<br>Energy: 1.587 eV<br>Osc.: 0.012	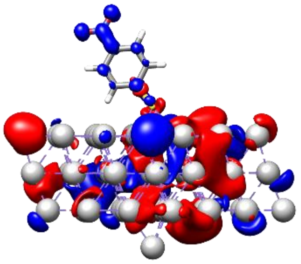<br>State 52<br>Energy: 1.627 eV<br>Osc.: 0.010	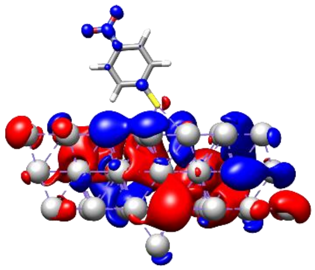<br>State 53<br>Energy: 1.640 eV<br>Osc.: 0.015
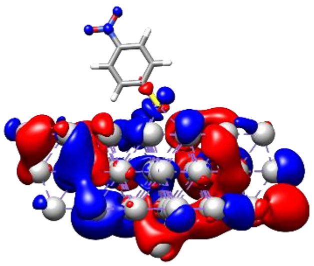<br>State 62<br>Energy: 1.814 eV<br>Osc.: 0.010	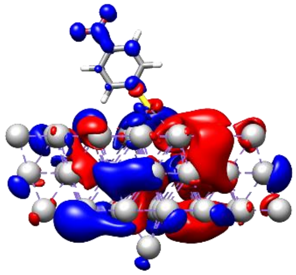<br>State 65<br>Energy: 1.846 eV<br>Osc.: 0.024	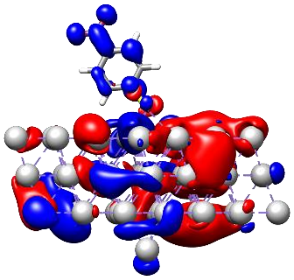<br>State 66<br>Energy: 1.865 eV<br>Osc.: 0.012	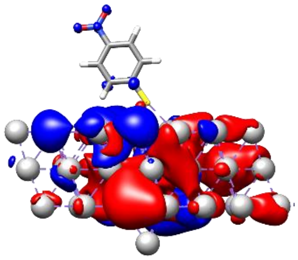<br>State 73<br>Energy: 1.933 eV<br>Osc.: 0.012
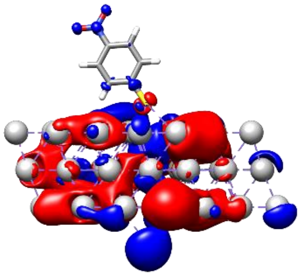<br>State 84<br>Energy: 2.084 eV<br>Osc.: 0.016	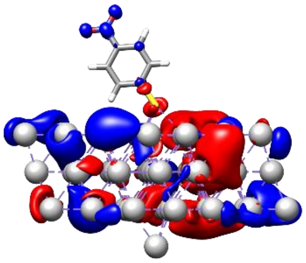<br>State 87<br>Energy: 2.132 eV<br>Osc.: 0.016	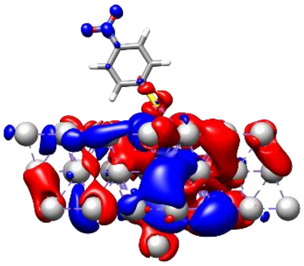<br>State 89<br>Energy: 2.160 eV<br>Osc.: 0.010	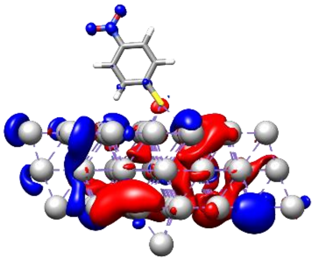<br>State 90<br>Energy: 2.171 eV<br>Osc.: 0.017

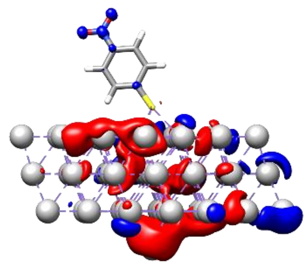<br>State 99<br>Energy: 2.280 eV<br>Osc.: 0.014	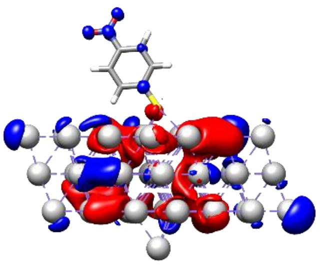<br>State 102<br>Energy: 2.302 eV<br>Osc.: 0.013	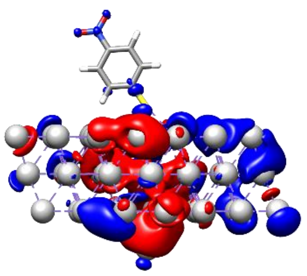<br>State 103<br>Energy: 2.313 eV<br>Osc.: 0.010	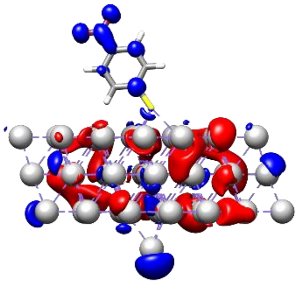<br>State 105<br>Energy: 2.334 eV<br>Osc.: 0.010
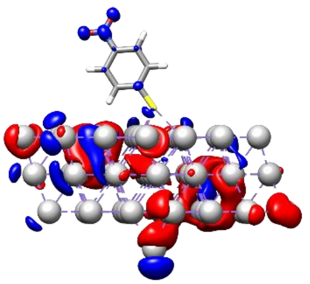<br>State 106<br>Energy: 2.357 eV<br>Osc.: 0.021	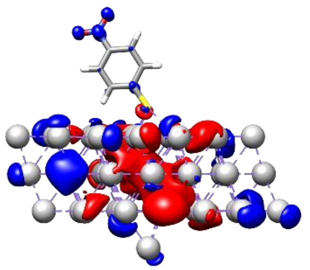<br>State 109<br>Energy: 2.383 eV<br>Osc.: 0.047	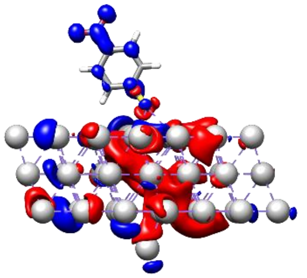<br>State 110<br>Energy: 2.398 eV<br>Osc.: 0.033	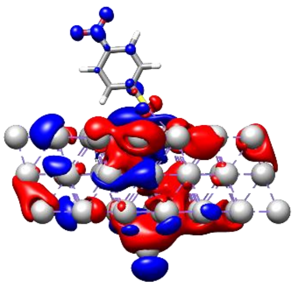<br>State 111<br>Energy: 2.404 eV<br>Osc.: 0.038
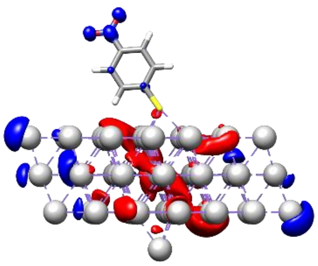<br>State 112<br>Energy: 2.433 eV<br>Osc.: 0.081	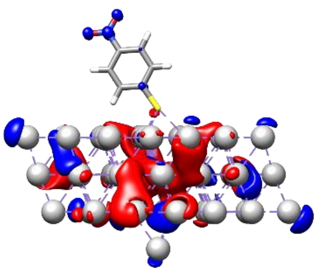<br>State 113<br>Energy: 2.447 eV<br>Osc.: 0.031	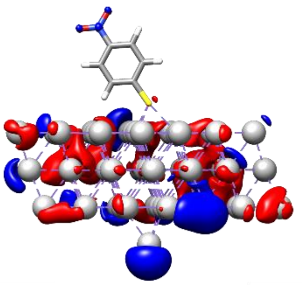<br>State 114<br>Energy: 2.452 eV<br>Osc.: 0.021	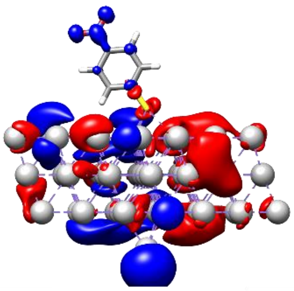<br>State 115<br>Energy: 2.455 eV<br>Osc.: 0.010
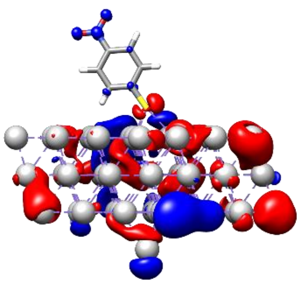<br>State 118<br>Energy: 2.488 eV<br>Osc.: 0.027	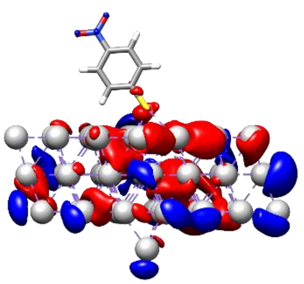<br>State 119<br>Energy: 2.498 eV<br>Osc.: 0.016	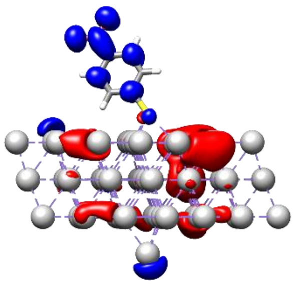<br>State 123<br>Energy: 2.530 eV<br>Osc.: 0.033	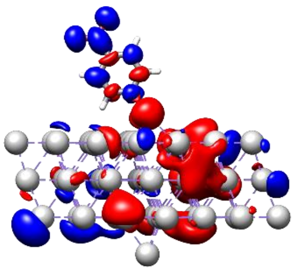<br>State 128<br>Energy: 2.580 eV<br>Osc.: 0.045

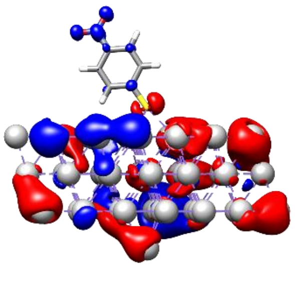<br>State 129<br>Energy: 2.589 eV<br>Osc.: 0.049	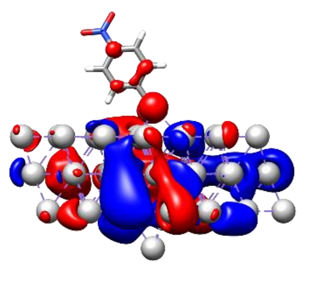<br>State 131<br>Energy: 2.600 eV<br>Osc.: 0.016	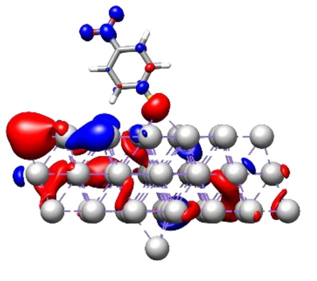<br>State 133<br>Energy: 2.624 eV<br>Osc.: 0.072	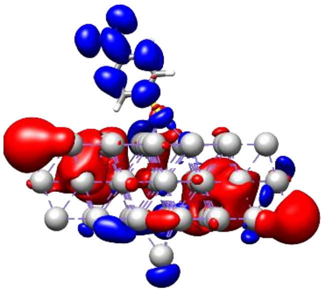<br>State 134<br>Energy: 2.628 eV<br>Osc.: 0.039
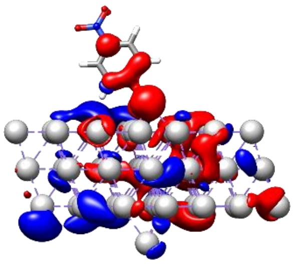<br>State 136<br>Energy: 2.638 eV<br>Osc.: 0.020	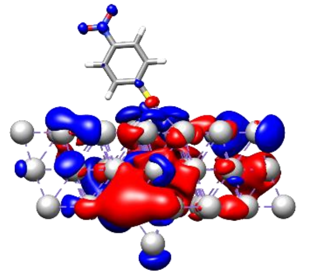<br>State 137<br>Energy: 2.651 eV<br>Osc.: 0.025	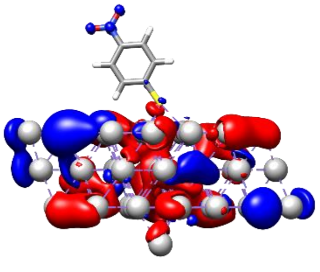<br>State 138<br>Energy: 2.654 eV<br>Osc.: 0.042	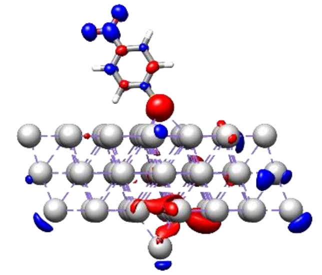<br>State 140<br>Energy: 2.667 eV<br>Osc.: 0.030
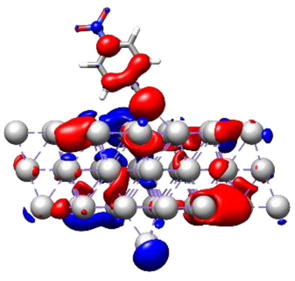<br>State 141<br>Energy: 2.671 eV<br>Osc.: 0.056	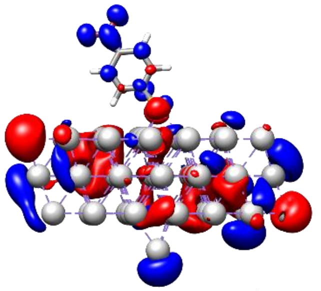<br>State 142<br>Energy: 2.686 eV<br>Osc.: 0.021	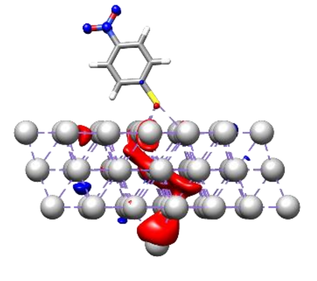<br>State 143<br>Energy: 2.696 eV<br>Osc.: 0.015	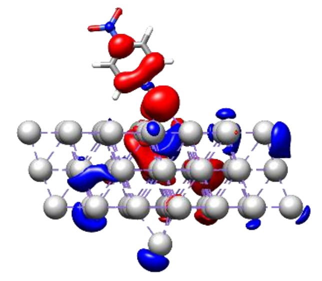<br>State 144<br>Energy: 2.701 eV<br>Osc.: 0.014
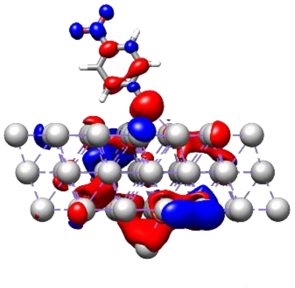<br>State 147<br>Energy: 2.735 eV<br>Osc.: 0.086	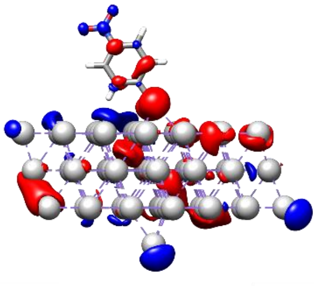<br>State 148<br>Energy: 2.742 eV<br>Osc.: 0.035	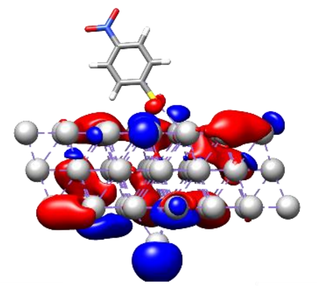<br>State 150<br>Energy: 2.751 eV<br>Osc.: 0.012	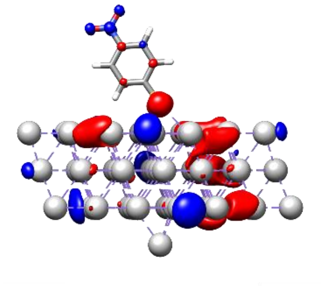<br>State 152<br>Energy: 2.776 eV<br>Osc.: 0.040

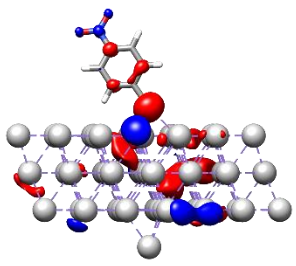

State 154
Energy: 2.795 eV
Osc.: 0.015

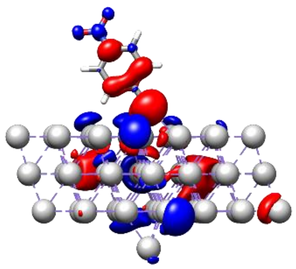

State 155
Energy: 2.802 eV
Osc.: 0.022

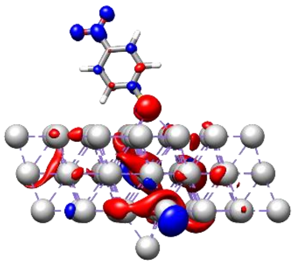

State 156
Energy: 2.811 eV
Osc.: 0.090

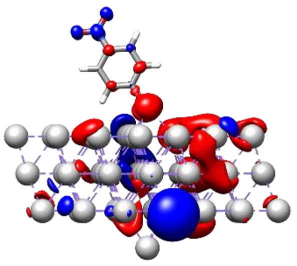

State 157
Energy: 2.817 eV
Osc.: 0.023

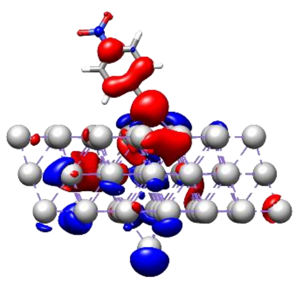

State 158
Energy: 2.830 eV
Osc.: 0.034

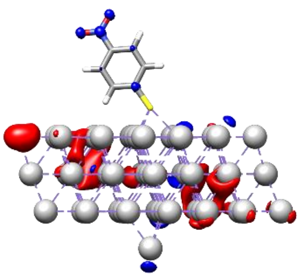

State 160
Energy: 2.840 eV
Osc.: 0.073

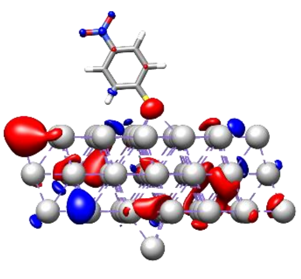

State 162
Energy: 2.855 eV
Osc.: 0.097

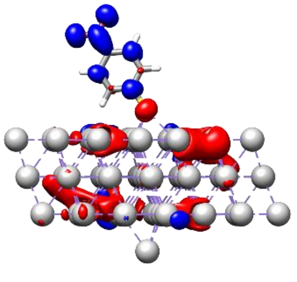

State 163
Energy: 2.863 eV
Osc.: 0.082

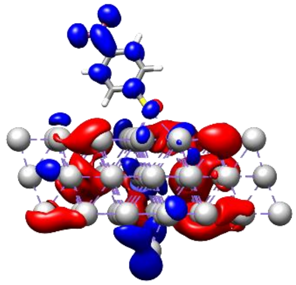

State 165
Energy: 2.885 eV
Osc.: 0.065

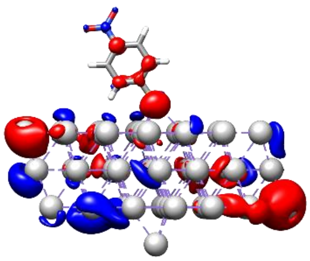

State 166
Energy: 2.894 eV
Osc.: 0.205

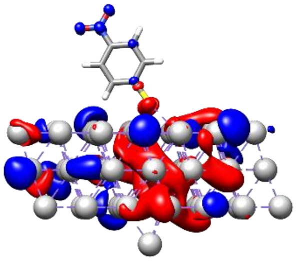

State 167
Energy: 2.903 eV
Osc.: 0.012

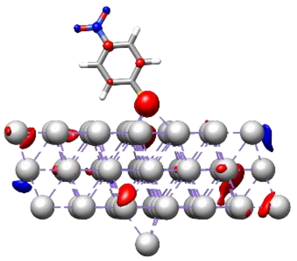

State 169
Energy: 2.917 eV
Osc.: 0.053

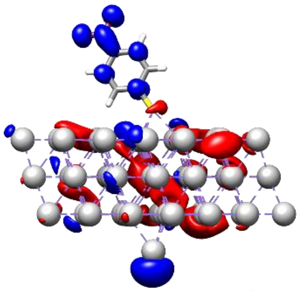

State 170
Energy: 2.923 eV
Osc.: 0.017

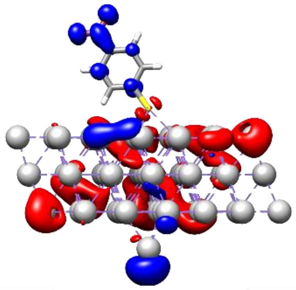

State 171
Energy: 2.928 eV
Osc.: 0.093

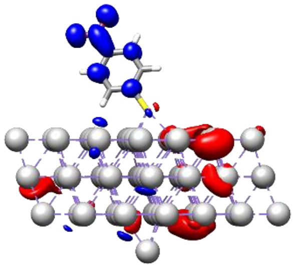

State 172
Energy: 2.939 eV
Osc.: 0.032

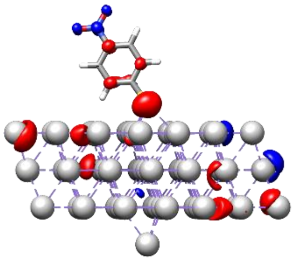

State 173
Energy: 2.947 eV
Osc.: 0.378

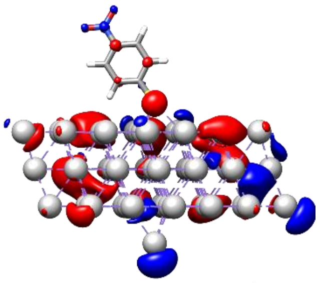<br>State 174<br>Energy: 2.952 eV<br>Osc.: 0.043	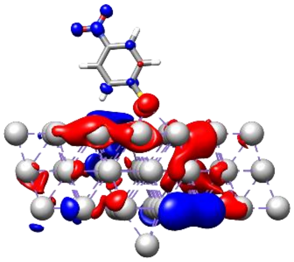<br>State 175<br>Energy: 2.955 eV<br>Osc.: 0.046	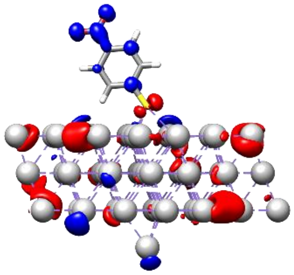<br>State 176<br>Energy: 2.965 eV<br>Osc.: 0.105	

Table S3: Charge density differences (CDDs) illustrating the nature of the low-lying dipole-allowed excitations of two 4-NBT on Au surface. Charge transfer takes place from red to blue.

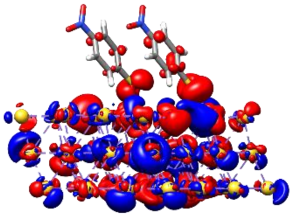<br>State 46<br>Energy: 1.580 eV<br>Osc.: 0.019	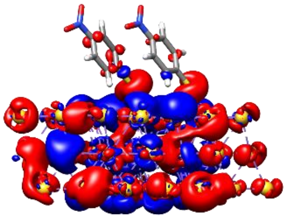<br>State 50<br>Energy: 1.660 eV<br>Osc.: 0.011	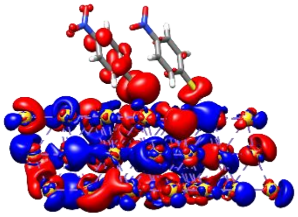<br>State 53<br>Energy: 1.708 eV<br>Osc.: 0.014	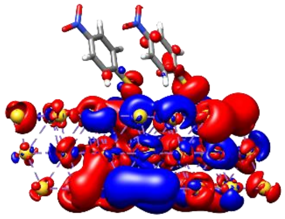<br>State 57<br>Energy: 1.769 eV<br>Osc.: 0.010
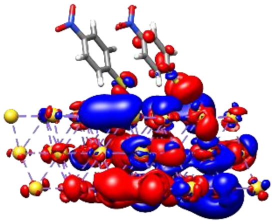<br>State 59<br>Energy: 1.795 eV<br>Osc.: 0.015	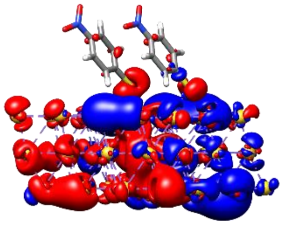<br>State 62<br>Energy: 1.841 eV<br>Osc.: 0.035	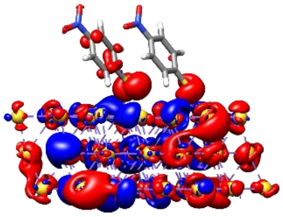<br>State 63<br>Energy: 1.873 eV<br>Osc.: 0.034	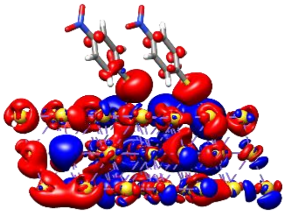<br>State 67<br>Energy: 1.925 eV<br>Osc.: 0.014

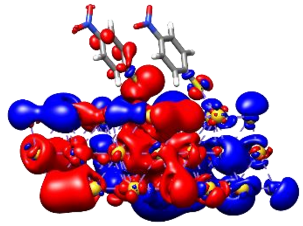<br>State 69<br>Energy: 1.941 eV<br>Osc.: 0.014	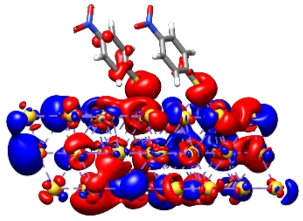<br>State 70<br>Energy: 1.970 eV<br>Osc.: 0.010	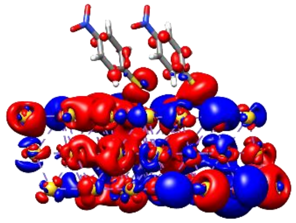<br>State 72<br>Energy: 2.002 eV<br>Osc.: 0.010	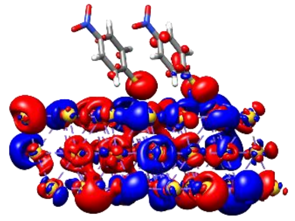<br>State 74<br>Energy: 2.032 eV<br>Osc.: 0.015
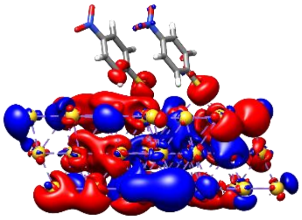<br>State 79<br>Energy: 2.088 eV<br>Osc.: 0.014	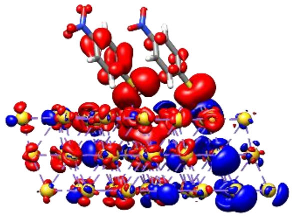<br>State 84<br>Energy: 2.157 eV<br>Osc.: 0.053	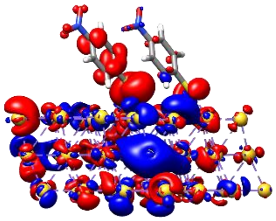<br>State 85<br>Energy: 2.174 eV<br>Osc.: 0.017	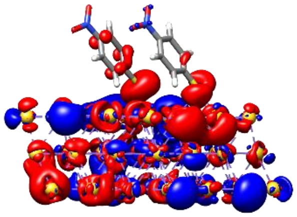<br>State 87<br>Energy: 2.207 eV<br>Osc.: 0.018
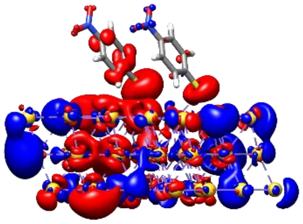<br>State 91<br>Energy: 2.248 eV<br>Osc.: 0.020	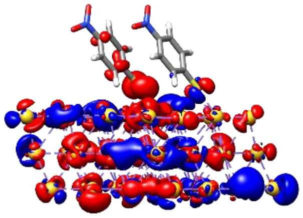<br>State 96<br>Energy: 2.312 eV<br>Osc.: 0.025	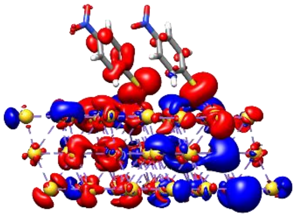<br>State 99<br>Energy: 2.352 eV<br>Osc.: 0.012	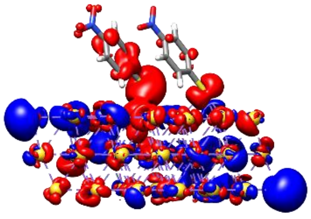<br>State 103<br>Energy: 2.404 eV<br>Osc.: 0.032

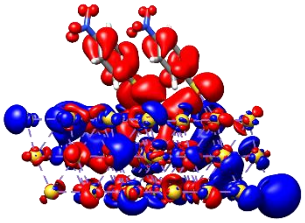<br>State 104<br>Energy: 2.411 eV<br>Osc.: 0.021	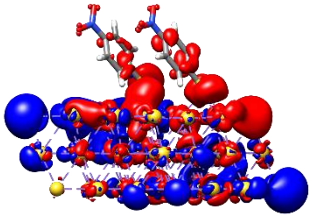<br>State 106<br>Energy: 2.442 eV<br>Osc.: 0.045	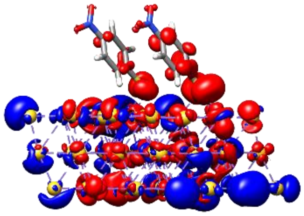<br>State 111<br>Energy: 2.484 eV<br>Osc.: 0.016	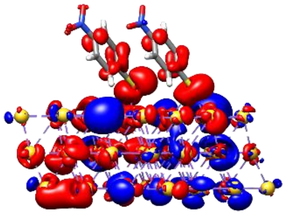<br>State 113<br>Energy: 2.501 eV<br>Osc.: 0.015
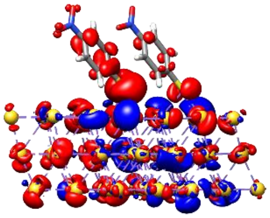<br>State 114<br>Energy: 2.512 eV<br>Osc.: 0.016	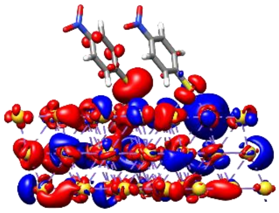<br>State 115<br>Energy: 2.522 eV<br>Osc.: 0.042	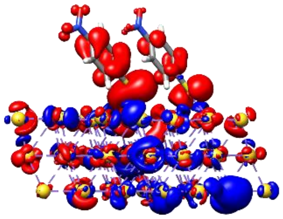<br>State 117<br>Energy: 2.538 eV<br>Osc.: 0.029	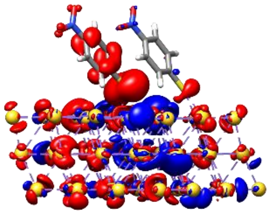<br>State 118<br>Energy: 2.546 eV<br>Osc.: 0.028
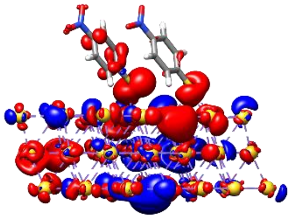<br>State 119<br>Energy: 2.559 eV<br>Osc.: 0.015	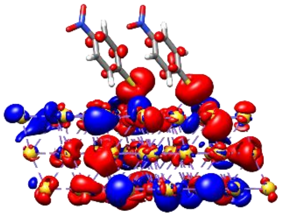<br>State 120<br>Energy: 2.562 eV<br>Osc.: 0.012	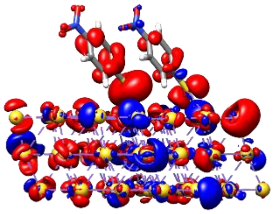<br>State 122<br>Energy: 2.585 eV<br>Osc.: 0.021	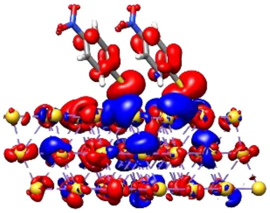<br>State 125<br>Energy: 2.616 eV<br>Osc.: 0.016

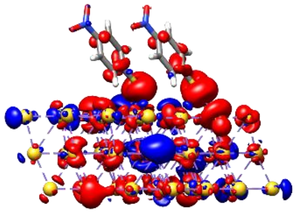<br>State 128<br>Energy: 2.649 eV<br>Osc.: 0.086	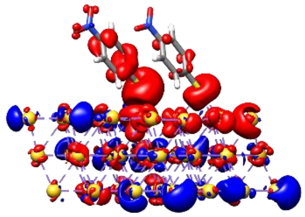<br>State 130<br>Energy: 2.675 eV<br>Osc.: 0.018	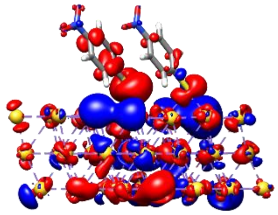<br>State 132<br>Energy: 2.696 eV<br>Osc.: 0.044	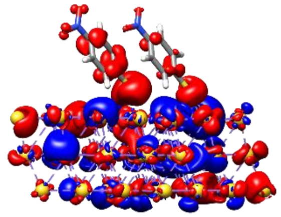<br>State 133<br>Energy: 2.698 eV<br>Osc.: 0.044
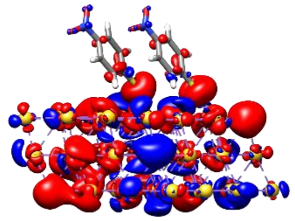<br>State 134<br>Energy: 2.717 eV<br>Osc.: 0.034	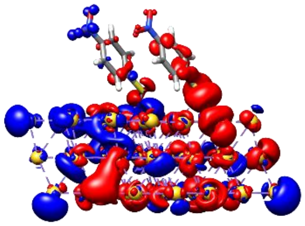<br>State 135<br>Energy: 2.723 eV<br>Osc.: 0.040	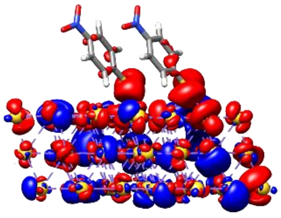<br>State 136<br>Energy: 2.731 eV<br>Osc.: 0.082	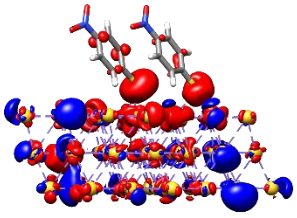<br>State 137<br>Energy: 2.737 eV<br>Osc.: 0.131
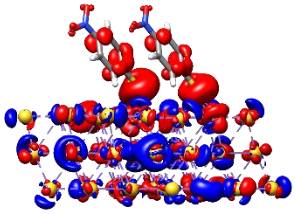<br>State 138<br>Energy: 2.745 eV<br>Osc.: 0.036	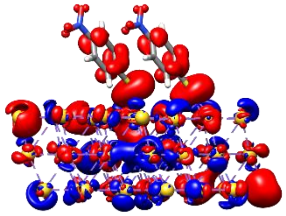<br>State 141<br>Energy: 2.770 eV<br>Osc.: 0.057	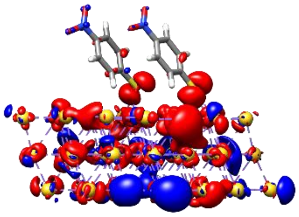<br>State 142<br>Energy: 2.774 eV<br>Osc.: 0.012	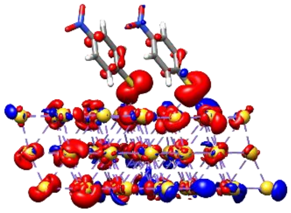<br>State 143<br>Energy: 2.790 eV<br>Osc.: 0.093

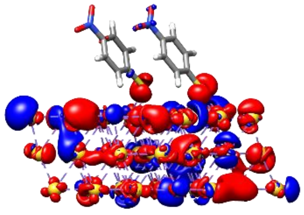<br>State 144<br>Energy: 2.812 eV<br>Osc.: 0.188	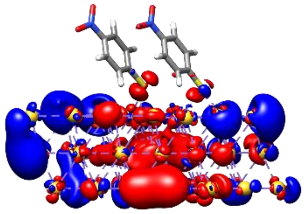<br>State 145<br>Energy: 2.819 eV<br>Osc.: 0.024	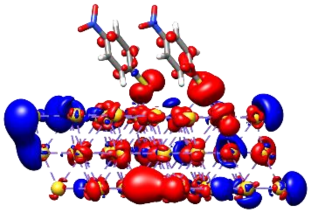<br>State 146<br>Energy: 2.823 eV<br>Osc.: 0.055	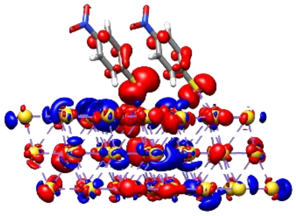<br>State 147<br>Energy: 2.831 eV<br>Osc.: 0.073
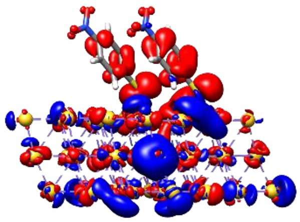<br>State 148<br>Energy: 2.838 eV<br>Osc.: 0.033	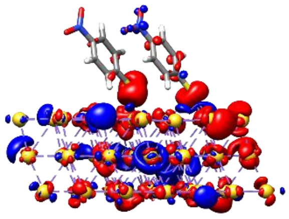<br>State 149<br>Energy: 2.846 eV<br>Osc.: 0.156	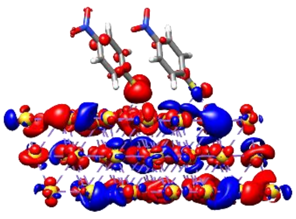<br>State 150<br>Energy: 2.855 eV<br>Osc.: 0.012	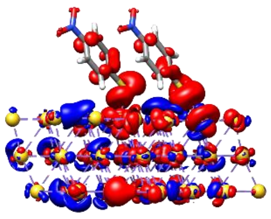<br>State 151<br>Energy: 2.857 eV<br>Osc.: 0.027
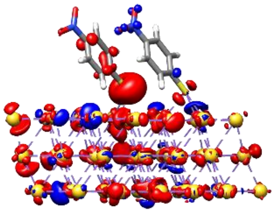<br>State 153<br>Energy: 2.880 eV<br>Osc.: 0.315	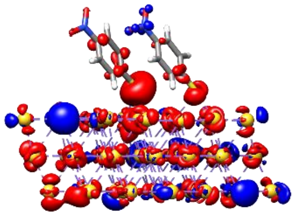<br>State 154<br>Energy: 2.891 eV<br>Osc.: 0.011	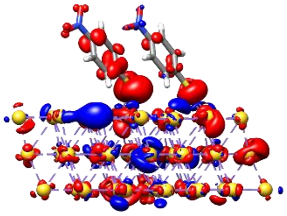<br>State 155<br>Energy: 2.898 eV<br>Osc.: 0.063	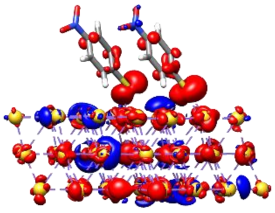<br>State 156<br>Energy: 2.911 eV<br>Osc.: 0.019

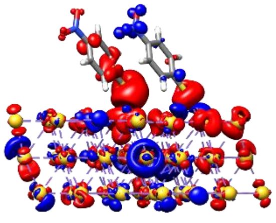<br>State 158<br>Energy: 2.923 eV<br>Osc.: 0.012	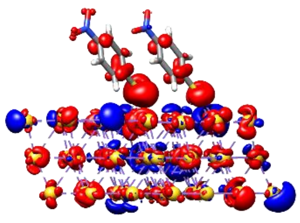<br>State 159<br>Energy: 2.931 eV<br>Osc.: 0.195	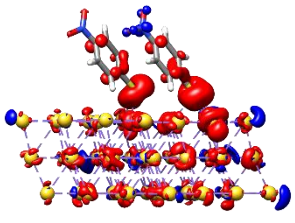<br>State 160<br>Energy: 2.932 eV<br>Osc.: 0.074	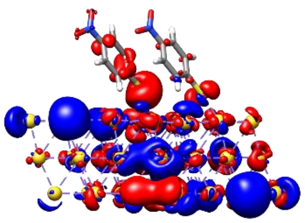<br>State 161<br>Energy: 2.940 eV<br>Osc.: 0.012
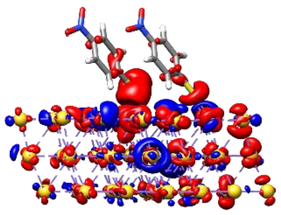<br>State 162<br>Energy: 2.949 eV<br>Osc.: 0.196	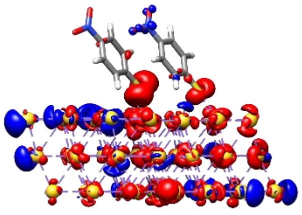<br>State 164<br>Energy: 2.962 eV<br>Osc.: 0.040	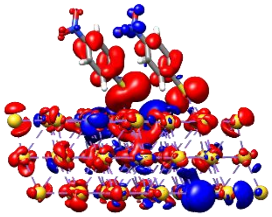<br>State 165<br>Energy: 2.966 eV<br>Osc.: 0.037	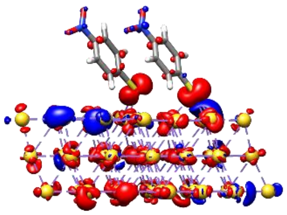<br>State 166<br>Energy: 2.976 eV<br>Osc.: 0.248
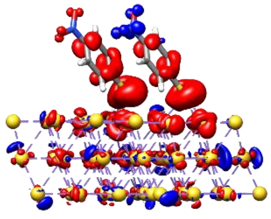<br>State 167<br>Energy: 2.991 eV<br>Osc.: 0.024	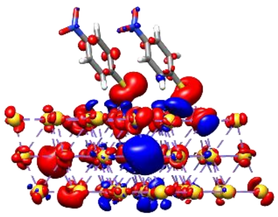<br>State 168<br>Energy: 2.995 eV<br>Osc.: 0.088	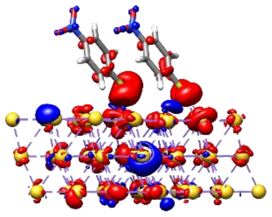<br>State 169<br>Energy: 3.003 eV<br>Osc.: 0.038	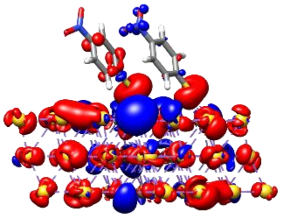<br>State 170<br>Energy: 3.006 eV<br>Osc.: 0.030
